# The IICR and the non-stationary structured coalescent: towards demographic inference with arbitrary changes in population structure

**DOI:** 10.1038/s41437-018-0148-0

**Published:** 2018-10-07

**Authors:** Willy Rodríguez, Olivier Mazet, Simona Grusea, Armando Arredondo, Josué M. Corujo, Simon Boitard, Lounès Chikhi

**Affiliations:** 1 Institut de Mathématiques de Toulouse, Université de Toulouse, Institut National des Sciences Appliquées, 31077 Toulouse, France; 2 Facultad de Matemática y Computación, Universidad de La Habana, La Havana, Cuba; 3 GenPhySE, Université de Toulouse, INRA, INPT, INP-ENVT, Castanet Tolosan, France; 4 Laboratoire Évolution & Diversité Biologique (EDB UMR 5174), Université de Toulouse Midi-Pyrénées, CNRS, IRD, UPS. 118 route de Narbonne, Bât. 4R1, 31062 Toulouse cedex 9, France; 5 Instituto Gulbenkian de Ciência, Rua da Quinta Grande, No. 6, P-2780-156 Oeiras, Portugal

## Abstract

In the last years, a wide range of methods allowing to reconstruct past population size changes from genome-wide data have been developed. At the same time, there has been an increasing recognition that population structure can generate genetic data similar to those produced under models of population size change. Recently, Mazet et al. (Heredity 116:362–371, 2016) showed that, for any model of population structure, it is always possible to find a panmictic model with a particular function of population size changes, having exactly the same distribution of *T*_2_ (the coalescence time for a sample of size two) as that of the structured model. They called this function IICR (Inverse Instantaneous Coalescence Rate) and showed that it does not necessarily correspond to population size changes under non-panmictic models. Besides, most of the methods used to analyse data under models of population structure tend to arbitrarily fix that structure and to minimise or neglect population size changes. Here, we extend the seminal work of Herbots (PhD thesis, University of London, 1994) on the structured coalescent and propose a new framework, the Non-Stationary Structured Coalescent (NSSC) that incorporates demographic events (changes in gene flow and/or deme sizes) to models of nearly any complexity. We show how to compute the IICR under a wide family of stationary and non-stationary models. As an example we address the question of human and Neanderthal evolution and discuss how the NSSC framework allows to interpret genomic data under this new perspective.

## Introduction

Reconstructing the demographic history of populations and species remains one of the great challenges of population genetics and statistical inference (Harpending and Rogers 2000; Beaumont et al. 2002; Goldstein and Chikhi 2002; Hey and Machado 2003; Li and Durbin 2011; Liu and Fu 2015; Scerri et al. 2018). In the last decades significant progress has been made in the development of likelihood and likelihood-free methods, hence facilitating the estimation of parameters of interest such as migration or admixture rates, and the dates of putative bottlenecks, expansions or splitting events (Beaumont 1999; Beaumont et al. 2002; Marjoram et al. 2003; Hey and Nielsen 2004; Gutenkunst et al. 2009; Li and Durbin 2011; Bunnefeld et al. 2015).

The rich body of methods and approaches that have been developed during that period can be divided into methods that ignore population structure and thus view the demographic history of species as a series of population size changes (Beaumont 1999; Chevalet and Nikolic 2010; Li and Durbin 2011; Liu and Fu 2015; Bunnefeld et al. 2015) and those that account for population structure (Nielsen and Wakeley 2001; Chikhi et al. 2001; Hey and Nielsen 2004; Gutenkunst et al. 2009; Gronau et al. 2011). In the first family of models, the number of population size changes can be fixed (Beaumont 1999) or it can be allowed to vary, hence allowing for more complex trajectories with a stepwise demographic history that can exhibit both expansions and contractions (Li and Durbin 2011; Nikolic and Chevalet 2014; Liu and Fu 2015; Boitard et al. 2016). In the second, the model of population structure is typically fixed *a priori* and relatively simple, and its parameters estimated (Chikhi et al. 2001; Hey and Nielsen 2004; Gutenkunst et al. 2009; Gronau et al. 2011). Some recent methods allow for complex multi-population split models with gene flow (Gutenkunst et al. 2009) or with admixture events (Gronau et al. 2011). However, while the sizes of ancestral and derived populations can be different in these methods, each one is usually assumed constant and the model structure usually remains fixed. If the evolutionary hypotheses made by the underlying models are violated (for example, if the populations evolve under a different type of structure), the estimated parameters may be difficult to interpret (Mazet et al. 2016; Chikhi et al. 2018).

There is thus no general inferential framework that allows the joint estimation of population structure and population size changes (Scerri et al. 2018). This is understandable because it would probably be beyond the current methods to estimate the parameters of such complex models. Still, if we wish to understand the recent evolutionary history of species, including that of humans, it may be necessary to identify the models with or without population structure that can (and those that cannot) explain patterns of genomic diversity.

This is challenging because an increasing number of studies have shown that population structure *per se* can generate spurious signals of population size change in genetic data. This suggests that the first group of methods may generate misleading histories of population size change (Wakeley 1999; Storz and Beaumont 2002; Chikhi et al. 2010; Heller et al. 2013; Mazet et al. 2016; Chikhi et al. 2018) that can explain the data as well or nearly as well as more realistic models of population structure. Since many species are *de facto* structured in space, a powerful approach to improve the inferential process might be to reduce the model and parameter space so as to focus on models that can explain the data in their genomic complexity. Models that cannot explain the data could then be rejected (Chikhi et al. 2018) or improved.

In the present study, we introduce a mathematical and conceptual framework based on the structured coalescent (Herbots 1994). We illustrate how the IICR curves could in principle be used to develop a powerful model choice and exclusion strategy. In a few words, the IICR is a time-dependent function that can be interpreted as an effective size in a panmictic population. However, for structured models this interpretation may be misleading. For instance, there are various IICR curves for the same demographic model that depend on the temporal and geographical sampling scheme (Mazet et al. 2016; Chikhi et al. 2018). For studies focusing on the impact of the sampling scheme on demographic inference, see also Wakeley (1999); Städler et al. (2009); Chikhi et al. (2010); Heller et al. (2013); Paz-Vinas et al. (2013). IICR curves can thus be seen as sample-dependent coalescent histories, which together may represent a unique signature for a complex model. The IICR is related to the PSMC method of Li and Durbin (2011) in the sense that the PSMC method, while generally interpreted in terms of population size changes, actually infers the IICR for a sample of size two (Mazet et al. 2016). The IICR curves can thus be seen as summaries of genomic information (Chikhi et al. 2018).

We extend previous work on the IICR by applying the theory of Markov chains (see for example Norris (1998)) to models of population structure of nearly any complexity (*i.e.*, including changes in gene flow and/or deme sizes). The idea of tracing back the ancestry of a sample using a continuous-time Markov chain has been discussed by a previous work (Hobolth et al. 2011). In this case authors computed the density of the coalescence times of two genes under an isolation with migration model (IM) and suggested that the coalescent under many structured models could be studied using a finite-state homogeneous continuous time Markov chain, in agreement with Herbots (1994). Here we go further and show how the transition rate matrices associated to a given structured model can be used to compute the corresponding IICR curves with very high accuracy, with a much lower computational time than the simulation-based approach used in Chikhi et al. (2018). We apply this new framework to the structured coalescent of Herbots (1994) and extend it to non-stationary models (*i.e.*, models in which parameters defining structure can change through time), hence introducing the Non-Stationary Structured Coalescent (NSSC), and discuss the possibility to extend it to less constrained genealogical models.

To that aim, we first review and summarise the main results and terminology required to link the Markov chain described by the structured coalescent with the notion of IICR. We acknowledge the seminal work of Herbots (1994) who derived the transition rate matrix corresponding to the structured coalescent. We apply this approach to compute the IICR of several models of population structure, such as the *n*-island model, and 1D and 2D stepping stone models, under arbitrary sampling schemes. Using the semi-group property we show how our results can be naturally extended to models with an arbitrary number of changes in gene flow. We then show how demes with different sizes (*e.g.*, continent-island models), or changes in the deme sizes can be easily incorporated into this framework. In addition, we show that transition rate matrices can be simplified using symmetries for several models (*n*-island, continent-island) reducing the computational costs by several orders of magnitude. We finally apply these results to humans and Neanderthals and identify models of population structure that can explain human and Neanderthal genomic diversity.

## The structured coalescent and transition rate matrices: towards the IICR

The distribution of coalescence times in models that account for population structure (*i.e.*, population subdivision) has been the centre of interest of important and early theoretical studies (Takahata 1988; Notohara 1990; Herbots 1994; Barton and Wilson 1995; Wakeley 1999, 2001; Nordborg 2001; Charlesworth et al. 2003). In particular, Herbots (1994) developed an elegant extension of the coalescent (Kingman 1982) for structured populations under a number of constraints regarding gene flow (see below). This extension, named structured coalescent, is based on a continuous-time Markov chain. It allows to compute explicitly the moment-generating function of the coalescence times under a wide range of models considering population structure (Herbots 1994; Wilkinson-Herbots 1998). In this section we review the terminology and theory leading to the structured coalescent, introduce transition rate matrices and show how they can be used to compute the IICR of Mazet et al. (2016).

### From the discrete-time model to the continuous-time approximation

Following Herbots (1994), we consider a haploid population divided into a finite number *n* of subpopulations or demes which are panmictic and whose size, *N*_*i*_ for deme *i*, is assumed to be large. Each deme is also assumed to behave as a haploid Wright–Fisher model. These demes are connected to each other by migration events. Every generation a proportion *q*_*ij*_ of the haploid individuals from deme *i* migrates to deme *j* (migrants are chosen without replacement, independently and uniformly from deme *i*). We assume that deme sizes and migration rates are constant in time. In this model the number of haploid individuals in deme *i* is *N*_*i*_ = 2*c*_*i*_*N*, where *c*_*i*_ is a positive integer and *N* is large. Also, the proportion *q*_*ij*_ is of the order of 1/*N* for every (*i*, *j*). In the classical *n*-island model of Wright (1931), the *c*_*i*_ are all identical and set to one. If we set $c: = \mathop {\sum}\nolimits_{i = 1}^n {\kern 1pt} c_i$, we can write the total haploid population size as *N*_*T*_ = 2*cN*. Note that in diploid applications, *c*_*i*_*N* is the number of diploid individuals in deme *i* and thus the diploid population size will be *cN*.

The structured coalescent of Herbots (1994) assumes that the size of each subpopulation is maintained constant under migration, which generates the following constraint at the population level:

1 $$\forall i,j:\quad c_i\mathop {\sum}\limits_{j \ne i} {\kern 1pt} q_{ij} = \mathop {\sum}\limits_{j \ne i} {\kern 1pt} c_jq_{ji},$$

where *q*_*ij*_ is the probability that one individual migrates from deme *i* to deme *j*. In other words, all outward migrants must be replaced by inward migrants from the other islands.

This condition is required by Herbots (1994) to prove the convergence to the structured coalescent when the population size goes to infinity. However, it is not required in the structured model of Notohara (1990) or when simulating data under structured models using the *ms* software of Hudson (2002). In a recent work Kozakai et al. (2016) gave a rigorous proof that the convergence to the structured coalescent holds even though condition (1) is violated. For simplicity, we will consider condition (1) in the models discussed below.

Looking now backward in time, Herbots (1994) defines the backward migration parameter from deme *i* to deme *j* (denoted *m*_*ij*_) as:

$$m_{ij} = \frac{{N_jq_{ji}}}{{N_i}} = \frac{{c_j}}{{c_i}}q_{ji}.$$

The backward migration parameter *m*_*ij*_ represents the proportion of individuals in deme *i* that were in deme *j* just before the migration step. Also, $m_i = \mathop {\sum}\nolimits_{i \ne j} {\kern 1pt} m_{ij}$ represents the proportion of individuals inside deme *i* that were in a different deme just before the migration step.

In this backward perspective, we suppose that we have a sample of *k* haploid genomes at a time which we arbitrarily call time zero. We then trace back the ancestral history of the *k* lineages until their MRCA (Most Recent Common Ancestor). We are interested in the statistical properties of the gene trees of this sample of *k* lineages at different loci in the genome. Following Herbots (1994), we define *α*_*N*_ := {*α*_*N*_(*r*);*r* = 0, 1, 2, …}, where *α*_*N*_(*r*) is a vector whose *i*^th^ component denotes the number of distinct lineages in subpopulation *i*, *r* generations ago.

Herbots (1994) proved that, measuring time in units of 2*N* generations, *α*_*N*_ converges to a continuous-time Markov chain called the structured coalescent, as *N* tends to infinity and as all *m*_*ij*_ (*i* ≠ *j*) tend to zero, in such a way that *M*_*ij*_/2 := 2*Nm*_*ij*_ and $M_i = \mathop {\sum}\nolimits_{j \ne i} {\kern 1pt} M_{ij}$ are constant, finite and non-zero. In the rest of the manuscript we drop the *N* index in *α*_*N*_, but we wish to stress that *α*_*N*_(*r*) represents the configuration of the remaining ancestral lineages at generation *r* backwards in the discrete-time model and *α*(*t*) represents the ancestral configuration *t* time units ago, in the continuous-time model. When *r* = 0 or *t* = 0, it is simply the initial sample configuration. The structured coalescent is thus the continuous-time Markov chain whose states are all the possible configurations for the ancestral lineages at different times in the past. It is thus characterised by the transition probabilities between configurations. A key element describing this Markovian process is its transition rate matrix denoted *Q* hereafter.

### The transition rate matrix of a continuous-time Markov chain

Transition rate matrices and the semigroup property are briefly introduced in this section. For a full background, see for instance Norris (1998). These notions are crucial because the semigroup property will be used to construct complex models, based on several consecutive simple ones, each represented by a different transition matrix. The semigroup property is related to the memoryless property of the exponential distribution. The semigroup property is concerned with Markov processes. It allows to start a new Markov process using the state reached from a previous Markov process. This section, while technical, explains how we will be able to change parameters of the structured models of interest in a very flexible manner. See below.

A transition rate matrix on the finite set *I* is a square matrix *Q* = *Q*(*i*, *j*), with *i*, *j* ∈ *I* satisfying the two following conditions:

- ∀*i* ≠ *j*, *Q*(*i*, *j*) ≥ 0,
- ∀*i*, $$Q(i,i) = - \mathop {\sum}\nolimits_{j \ne i} {\kern 1pt} Q(i,j)$$.

If we now define, for all *t* ≥ 0, the exponential matrix *P*_*t*_ = *e*^*tQ*^ which has the same size as *Q*, *P*_*t*_ then satisfies the following properties, for all *s*, *t*:

- *P*_*t*+*s*_ = *P*_*t*_*P*_*s*_ (semigroup property),
- $$P_t^\prime = \frac{{\mathrm{d}}}{{{\mathrm{d}}t}}P_t = QP_t = P_tQ$$,
- *P*_*t*_ is a stochastic matrix (each coefficient is non-negative and the sum over each row is one). Also each coefficient of the matrix *P*_*t*_, for all *t*≥0, is a transition probability:
  $$P_t(i,j) = {\Bbb P}(X_t = j|X_0 = i),$$
  where (*X*_*t*_)_*t*≥0_ is a continuous-time Markov chain on the finite set *I*. In other words, *X*_*t*_ is a jump process, whose behaviour is the following:
- if at a given time *s* ≥ 0 we have *X*_*s*_ = *i*, then it jumps away from state *i* after an exponential time of parameter −*Q*(*i*, *i*), which does not depend on *s*,
- at each jump from state *i*, the rate at which state *j* is reached is ${\textstyle{{Q(i,j)} \over {\mathop {\sum}\nolimits_{j \ne i} {\kern 1pt} Q(i,j)}}}$ = −*Q*(*i*, *j*)/*Q*(*i*, *i*).

The transition rate matrix *Q* then contains all the information on the behaviour of (*X*_*t*_)_*t*≥0_, given the initial condition *X*_0_. We can see that, for all *i* ∈ *I*, the parameter *Q*(*i*, *j*) is the rate of going from *i* to *j*, as soon as *j* ≠ *i*, and the parameter −*Q*(*i*, *i*) is the rate of leaving *i*.

In the case of the structured coalescent the jump process of interest is the ancestral lineage process. The set *I* is the set of possible configurations *α* = (*α*_1_, …, *α*_*n*_), where *α*_*i*_ is the number of lineages present in the *i*^th^ deme, and *n* the number of demes. A ‘jump’ between two configurations occurs when a lineage migrates from one deme to another (say, from deme *i* to deme *j*), or when a coalescence takes place within a deme in which there are at least two lineages. We thus have now all the elements necessary to compute the IICR for stationary models under the structured coalescent.

## Transition rate matrices allow us to compute the IICR for a wide family of structured models

Mazet et al. (2016) introduced and defined the IICR. They derived it analytically for the *n*-island model and for *k* = 2 lineages for the only two distinguishable sampling schemes (the two lineages in the same deme, respectively in different demes) available for that model (initial configurations or states of the Markov chain). In this section we show how transition rate matrices can be used to analyse a wide family of models of population structure. We take the case of *k* = 2 and step by step explain how the transition rate matrix can be constructed. We then describe the general algorithm used to construct the IICR for all the models analysed here for *k* = 2. We finally apply this method to the *n*-island model and show that we can re-derive the results obtained by Mazet et al. (2015) and Herbots (1994).

### General case

As noted above, Herbots’ discrete-time process converges to a continuous-time Markov process (called structured coalescent). Here we describe in more details how to construct the associated transition rate matrix. Let us assume that we have numbered the demes of the model from 1 to *n* where *n* is the total number of demes. Then the vector (*c*_1_, *c*_2_, …, *c*_*n*_) indicates the size of each deme. We take a sample of *k* genes (*k* ≥ 2) from the population at the present (*t* = 0) and we trace the ancestral lineages back to the MRCA. The vector *α* = (*α*_*i*_)_1≤*i*≤*n*_, where *α*_*i*_ is the number of ancestral lineages in deme *i*, represents a possible ancestral configuration for the lineages when going backwards in time. For example, for *n* = 3 demes and *k* = 2 samples, the vector *α* = (1, 1, 0) is an element of ${\Bbb N}^3$ and indicates that there is one ancestral lineage in deme 1, one ancestral lineage in deme 2 and no ancestral lineage in deme 3. Note that $k = \left| \alpha \right| = \mathop {\sum}\nolimits_{i = 1}^n {\kern 1pt} \alpha _i$. We call *E*_*k*,*n*_ the set of all possible states of a structured model with *n* demes and a sample of size *k*. We have:

$$E_{k,n} = \left\{ {\alpha ,\alpha \in {\Bbb N}^n,1 < \left| \alpha \right| \le k} \right\} \cup \{ c\}$$

where *c* represents the state when the MRCA of the sample is reached (|*α*| = 1).

The Markov chain can change from one state *α* ∈ *E*_*k*,*n*_ to another state *β* ∈ *E*_*k*,*n*_ either by a migration event (which implies that |*β*| = |*α*|) or by a coalescence event inside a deme (which implies that |*β*| = |*α*| − 1). Before constructing the associated transition rate matrix we need to define an order on *E*_*k*,*n*_. We choose the inverse lexicographical order. For example for *n* = 3 and *k* = 2 it would be:

$$(2,0,0) \prec (1,1,0) \prec (1,0,1) \prec (0,2,0) \prec (0,1,1) \prec (0,0,2) \prec c.$$

Note that the state *c* (when the MRCA of the sample is reached) is placed in the last position. We denote *ϕ* the function that associates an element of *E*_*k*,*n*_ with the corresponding index in the inverse lexicographical order. For example, taking the previous example, for *α* = (2, 0, 0) it will be *ϕ*(*α*) = 1 and *ϕ*(*c*) = 7. Throughout the next sections we will assume that there is an order on the set *E*_*k*,*n*_ given by the function *ϕ*. We define *n*_*α*_ := *ϕ*(*α*) so that *P*_*t*_(*n*_*α*_, 1) refers to the first element of the row *n*_*α*_ in the matrix *P*_*t*_.

The corresponding transition rate matrix can be constructed as:

2 $$Q\left( {n_\alpha ,n_\beta } \right) = \left\{ {\begin{array}{*{20}{c}} {\alpha _i\frac{{M_{ij}}}{2}} & {{\mathrm{if}}\,\beta = \alpha - \epsilon ^i + \epsilon ^j\quad (i \ne j)} \\ {\frac{1}{{c_i}}\frac{{\alpha _i(\alpha _i - 1)}}{2}} & {{\mathrm{if}}\,\beta = \alpha - \epsilon ^i} \\ { - \mathop {\sum}\limits_i \left( {\alpha _i\frac{{M_i}}{2} + \frac{1}{{c_i}}\frac{{\alpha _i(\alpha _i - 1)}}{2}} \right)} & {{\mathrm{if}}\,\beta = \alpha } \\ 0 & {{\mathrm{otherwise}},} \end{array}} \right.$$

where $\epsilon ^i$ is the vector whose components are 1 on the *i*^th^ position and 0 elsewhere.

The matrix *Q* describes two types of possible events for each configuration *α*:

- $\beta = \alpha - \epsilon ^i + \epsilon ^j$ when one lineage migrates (backward in time) from island *i* to island *j*. The rate of this migration is *M*_*ij*_/2 (migration rate to deme *j* for each lineage in deme *i*) times *α*_*i*_, the number of lineages present in deme *i*.
- $\beta = \alpha - \epsilon ^i$ denotes a coalescence event between two lineages in deme *i*, which reduces the number of lineages by one in this deme. This occurs only if *α*_*i*_ ≥ 2. If this is not the case we can see that *α*_*i*_(*α*_*i*_ − 1) = 0. The term *α*_*i*_(*α*_*i*_ − 1)/2 is the number of possible pairs among the *α*_*i*_ lineages. This term is multiplied by 1/*c*_*i*_ since the *i*^th^ island has a population size equal to 2*c*_*i*_*N*, and 1/*c*_*i*_ is the coalescence rate for each pair of lineages in this island since time is scaled by 2*N*.

Since no other kind of event can occur than a migration or a coalescence, and multiple coalescences or migrations are negligible, the other rates are null. Note that the opposite of the diagonal coefficient −*Q*(*n*_*α*_, *n*_*α*_) is the total jump rate from configuration *α*.

The transition rate matrix can be very large depending on the model of population structure assumed and on the sample size. For *k* ≤ *n* the number of states is on the order of *n*^*k*^, and the matrix will have on the order of *n*^2*k*^ terms.

### Case of a sample of two lineages (*k* = 2)

We now consider the case where we take a sample of two lineages (*i.e.*, *k* = 2 corresponding to two haploid genomes or one diploid genome) in an arbitrary model of population structure with *n* demes of size 2*c*_*i*_*N*, for large *N*. We can reduce all possible configurations to only two types of configurations, excluding the configuration where the two lineages have coalesced:

- both lineages are in the same deme *i*: $\alpha = 2\epsilon ^i$,
- the two lineages are in different demes, say, demes *i* and *j* with *i* ≠ *j*: $\alpha = \epsilon ^i + \epsilon ^j$.

When the two lineages are in the same deme (first case), there are two possible events that can change the configuration: a coalescence with rate 1/*c*_*i*_, or a (backward) migration from *i* to *j* ≠ *i* for each lineage, with rate *α*_*i*_*M*_*ij*_/2 for both lineages, hence a total rate of *α*_*i*_*M*_*ij*_. When a coalescence happens, the number of lineages decreases by one. When a migration from deme *i* to deme *j* happens, the new configuration is one in which the lineages are now in different demes, which is a second-type configuration.

When the two lineages are in different demes, no coalescence can occur and the two lineages may either stay in the same deme or migrate to another deme, from *i* to $\ell$ (which can be equal to *j*) for the first lineage, with rate $\alpha _iM_{i\ell }{\mathrm{/}}2$, or from *j* to $\ell$ (which can be equal to *i*) for the second lineage, with rate $\alpha _jM_{j\ell }{\mathrm{/}}2$. If the lineages end up in the same deme we are back to a configuration of the first type, otherwise, we end up in a second-type configuration.

By definition, the number of rows and columns of the full transition rate matrix (that we will call *n*_*c*_) is the number of different configurations for the ancestral lineages. In the case of a model with *n* demes and a sample of size *k* = 2, we have that *n*_*c*_ = *n*^2^ + 1. We will assume that the “last configuration” is the one in which the two lineages have coalesced, and thus ignore where the coalescence took place. Also note that the rate of a coalescence event in deme *i* (which is equal to 1/*c*_*i*_) depends on the size of deme *i*. In the transition rate matrices that we will use here the coalescence configuration corresponds to the last row and column. In the Supplementary Materials we give a general algorithm that can be used to construct the transition rate matrix.

### Using the transition rate matrix to derive the distribution of coalescence times and evaluate the IICR for samples of size two

We now focus on the coalescence time between two lineages and see that we can derive the IICR in terms of transition rate matrices. The theory of Markov chains (Norris 1998) gives the tools allowing to compute the probability distribution of *T*_2_ based on the matrix exponential of the transition rate matrix for the model of interest

$$P_t = e^{tQ},$$

where *P*_*t*_ is the transition semigroup of the corresponding Markov process, *i.e.*, $P_t\left( {n_\alpha ,n_\beta } \right)$ = ${\Bbb P}(\alpha (t) = \beta |\;\alpha (0) = \alpha )$, where *α*(*t*) denotes the ancestral lineages configuration at time *t* in the past and *α*(0) represents the initial sample configuration.

As noted in Section ‘The transition rate matrix of a continuous-time Markov chain’, the terms of *P*_*t*_ represent the transitions probabilities of interest. For instance, the term in row *n*_*α*_ and column *n*_*β*_ of *P*_*t*_ represents the probability that the process is in the configuration *β* at time *t* given that it was in the configuration *α* at time zero. Thus, the probability that two lineages in the configuration *α* at *t* = 0 have reached their most recent common ancestor at time *t* can be found as *P*_*t*_(*n*_*α*_, *n*_*c*_), where *n*_*c*_ is the last column since *n*_*c*_ = *ϕ*(*c*) is the column number of the coalescence state.

Consequently, if we denote by $T_2^\alpha$ the coalescence time of two lineages sampled in the configuration *α*, the cumulative distribution function (*cdf*) of this random variable can be computed from the transition semigroup:

$$F_{T_2^\alpha }(t) = {\Bbb P}\left( {T_2^\alpha \le t} \right) = P_t\left( {n_\alpha ,n_c} \right).$$

The probability density function (*pdf*) of $T_2^\alpha$, $f_{T_2^\alpha }(t)$, is by definition the derivative of $F_{T_2^\alpha }(t)$. It can thus be computed from the matrix *P*_*t*_, by using the property

$$P_t^\prime = P_tQ = QP_t,$$

where $P_t^\prime$ is the matrix whose cells contain the derivative of the corresponding cells of *P*_*t*_. We can thus write

$$f_{T_2^\alpha }(t) = \frac{d}{{dt}}{\Bbb P}\left( {T_2^\alpha \le t} \right) = P_t^\prime \left( {n_\alpha ,n_c} \right) = \left( {P_tQ} \right)\left( {n_\alpha ,n_c} \right).$$

For any time *t* ≥ 0, the instantaneous coalescence rate is (Mazet et al. 2016) the ratio

$$\frac{{f_{T_2^\alpha }(t)}}{{1 - {\Bbb P}\left( {T_2^\alpha \le t} \right)}}.$$

The Inverse Instantaneous Coalescence Rate (IICR) of Mazet et al. (2016), is simply the inverse of this ratio, in which all the terms can be written as a function of *P*_*t*_ and the transition rate matrix, namely:

$${\mathrm{IICR}}(t) = \frac{{1 - P_t\left( {n_\alpha ,n_c} \right)}}{{\left( {P_tQ} \right)\left( {n_\alpha ,n_c} \right)}}.$$

In the next section, we show how transition rate matrices can be used to re-derive the analytical results of Mazet et al. (2016) on the IICR of the *n*-island model.

### The IICR of the ***n***-island model for *k* = 2 using the simplified transition rate matrices

**Fig. 1:**
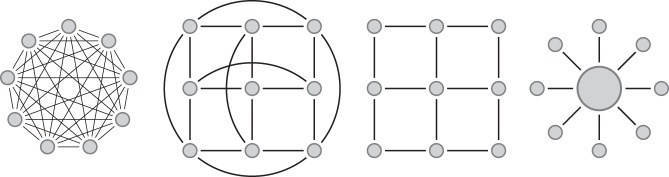
Diagrams for commonly used structured models. From left to right: *n*-island, torus 2D stepping stone, 2D stepping stone and continent-island model

In the symmetric island model of Wright (1931) the *n* demes (*n* ≥ 2) are equal-sized islands with the same migration rate between any two islands (Fig. 1). With the notations above, we have ∀*i* = 1, …, *n*, *c*_*i*_ = 1, *M*_*i*_ = *M* and *M*_*ij*_ = *M*/(*n* − 1) for *j* ≠ *i*. Taking into account the fact that the model is fully symmetrical, we only need to consider two configurations for a sample of two lineages: they are either in the same deme (denoted *s*) or in different demes (denoted *d*). There is a third state that corresponds to the coalescence event which takes place at rate 1. We thus obtain the simplified transition rate matrix

$$Q = \left( {\begin{array}{*{20}{c}} { - 1 - M} & M & 1 \\ {\frac{M}{{n - 1}}} & { - \frac{M}{{n - 1}}} & 0 \\ 0 & 0 & 0 \end{array}} \right),$$

where the first configuration is *s*, the second is *d*, and the third one corresponds to a coalescence event, which can only occur when both lineages are in the same island.

This matrix is simple and small enough to allow the derivation of explicit formula for its exponential *P*_*t*_ = *e*^*tQ*^ and hence for the corresponding IICR functions under the two possible starting configurations (IICR_s_ or IICR_d_ for samples taken in the same or different demes, respectively):

$${\mathrm{IICR}}_{\mathrm{s}}(t) = \frac{{1 - P_t(1,3)}}{{(P_tQ)(1,3)}} = \frac{{(1 - \beta )e^{ - \alpha t} + (\alpha - 1)e^{ - \beta t}}}{{(\alpha - \gamma )e^{ - \alpha t} + (\gamma - \beta )e^{ - \beta t}}}$$

and

$${\mathrm{IICR}}_{\mathrm{d}}(t) = \frac{{1 - P_t(2,3)}}{{(P_tQ)(2,3)}} = \frac{{\beta e^{ - \alpha t} - \alpha e^{ - \beta t}}}{{\gamma e^{ - \alpha t} - \gamma e^{ - \beta t}}},$$

with

$$\begin{array}{l}\alpha = \frac{1}{2}\left( {1 + n\gamma + \sqrt {\mathrm{\Delta }} } \right),\\ \beta = \frac{1}{2}\left( {1 + n\gamma - \sqrt {\mathrm{\Delta }} } \right),\\ {\mathrm{\Delta }} = (1 + n\gamma )^2 - 4\gamma ,\\ \gamma = \frac{M}{{n - 1}} = \alpha \beta .\end{array}$$

**Fig. 2:**
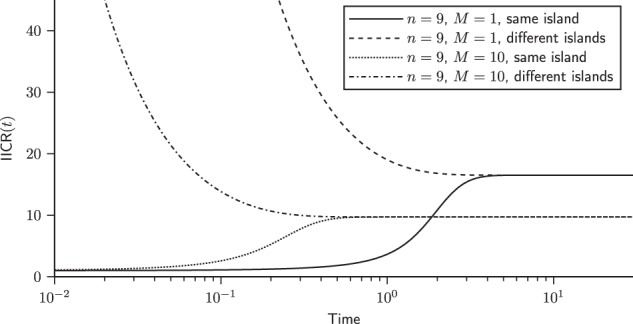
IICR for the *n*-island model. We plotted the IICR for a model with *n* = 9 islands and assuming two values for the migration rate, *M* = 1 and *M* = 10. For each model we started with the two configurations in which the genes are either sampled in the *same* (IICR_s_) or in *different* (IICR_d_) islands

These formulae are identical to those of Mazet et al. (2015), who obtained them using a different approach. We can see the plots of the IICR_s_ and IICR_d_ for the *n*-island model in Fig. 2.

As discussed in Mazet et al. (2016), the IICR curves observed in Fig. 2, may lead to the misleading interpretation that there was a recent decrease in population size when the two haploid genomes are sampled in the same deme of a structured population. This is because when we sample genes in the same deme, the coalescence rate in the recent past is mainly driven by the size of the local deme. However, as time increases backward a migration event may occur, which then will significantly change the rate at which coalescence events take place. Indeed, a coalescent event is only possible when the two lineages are in the same deme (which is only possible after at least one more migration event). This change in coalescent rates that significantly decrease as times goes backward, explains why a bottleneck signal is observed. When genes are sampled in different demes the opposite effect is observed. Since they cannot coalesce until they are present in the same deme, the instantaneous coalescence rate is zero at the time of sampling (*t* = 0) and close to zero for small *t* values, which corresponds to a population of infinite or very large size. This explains why the IICR curves suggest a recent population increase, with time going forward. These results have been derived analytically by Mazet et al. (2016) for the *n*-island model, and confirmed by simulation for several models of structured populations by Chikhi et al. (2018). While these bottleneck and expansion signals are particularly important when the migration rate is low, the above reasoning is also valid for high values of migration rate (high *M*). It is also important to stress that the analytical results of Mazet et al. (2016) hold for any value of *M*. However, when *M* increases the effect of population structure decreases as expected. For instance, under a stationary model, and for the *IICR*_*s*_ the apparent decrease in population size becomes increasingly closer to the recent past (Mazet et al. 2016), and may thus be less and less detectable with genetic data. In other words, when *M* increases the *n*-island model looks increasingly like a panmictic population, whether the two haploid genomes are sampled in the same or in different demes.

## Constructing the IICR for two stationary models, the 2D stepping stone and continent-island models

We now apply the framework and algorithm described above to two stationary models. To our knowledge, there is no analytical expression for the distribution of the coalescence time *T*_2_ under these two models. The transition rate matrices and IICR results for several other stationary models are shown in the Supplementary Materials.

### 2D stepping stone models with and without edges

Stepping stone models (Kimura 1953; Malécot and Blaringhem 1948) assume that the demes are located at the nodes of a regular lattice in one or two dimensions (hereafter 1D and 2D stepping stone models). Each deme can have up to four neighbours and migration events are only possible between neighbouring demes. These models incorporate space, and are thus thought to be more realistic than the *n*-island model described above, which implicitly assumes that migration is as likely between neighbouring than between distant islands. The border demes can either be connected with each other, hence forming a torus, or can behave as bouncing borders (Fig. 1). In some models the bouncing borders migrants are assumed to stay in their deme, whereas in other models they are distributed among the demes to which their deme is connected.

For the 2D stepping stone model, we set, ∀*i*, *j* = 1, …, *n*, *c*_*i*_ = 1 and *M*_*ij*_ = *M*/4 if islands *i* and *j* are neighbours, and *M*_*ij*_ = 0 otherwise. The difference between the models with and without edges used here is thus in the way neighbours are defined. In the model with borders the four corner islands have only two neighbours, the islands on the borders of the lattice have three, and the others have four neighbours (see Fig. 1).

**Fig. 3:**
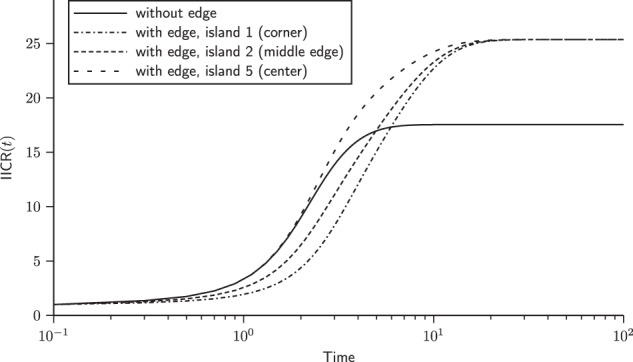
IICR plots for the 2D stepping stone model. Here we assumed a model with 3 × 3 = 9 islands and *M* = 1, with and without edge effect. In the model with edge effect, we plot the three ways to sample two lineages in the same island: in island 1, 3, 7 or 9 (corner), in island 2, 4, 6 or 8 (middle of the edge), and in island 5 (center of the lattice)

Figure 3 shows the IICR_s_ (two haploid genomes sampled in the same deme, or one diploid genome), for a 3 × 3 stepping stone model with and without borders (Fig. 1). In the latter case (no borders), all demes are statistically identical, and there can thus be only one IICR_s_ plot. In the model with borders, there are three possible ways to sample a diploid individual, and three IICR_s_ are plotted. This figure confirms the results of Chikhi et al. (2018) by showing that the IICR_s_ plots for a stepping stone are also S-shaped. They all start in the recent past at a value equal to the deme size and converge in the ancient past towards the same plateau. However, it is remarkable that they differ in the trajectory from the present to the plateau value, depending on the location of the deme (corner, border or centre). These results thus confirm that in a stepping stone model, two diploid individuals sampled in different demes (*i.e.*, geographical regions) will both exhibit signals of population decrease that will be different even though the population size was constant and they both belonged to the same structured model (Chikhi et al. 2018). Note that, as for the *n*-island model, the IICR exhibits a signal of spurious population increase when the two genes are sampled in different demes (IICR_d_, see Supplementary Materials).

### Continent-island model

#### General case

Here we assume a model where the population is divided into *n* demes (one big deme called continent and *n* − 1 equally sized demes, smaller than the continent, called islands). The continent is connected with the remaining *n* − 1 islands, but the islands are not connected between each other (Fig. 1). Therefore, migration can only occur between the continent and the islands, but not between different islands. Note that the continent-island model described here differs from the standard definition which assumes that the backward migration from the islands to the continent is zero, or negligible. This would break constraint 1 but the process would still converge to the structured coalescent (Kozakai et al. 2016). To analyse our model we order the *n* demes in such a way that the continent is deme number 1, whose (scaled) size is *c*_1_. We denote *c*_2_ the size of the other islands, and *M*_1_/2 the (scaled) migration rate from the continent to each island, and *M*_2_/2 the migration rate from each island to the continent. Condition (1) implies that we have the following constraint:

3 $$c_1\left( {(n - 1)\frac{{M_1}}{2}} \right) = \left( {(n - 1)c_2} \right)\frac{{M_2}}{2} \Leftrightarrow \frac{{c_1}}{{c_2}} = \frac{{M_2}}{{M_1}}.$$

For the case *n* ≥ 3, the symmetry of the model allows us to consider, for a sample of two lineages, only five possible different configurations:

1. Both lineages are in the continent. A coalescence can occur with rate 1/*c*_1_, leading to configuration 5, or any of the two lineages may migrate to one of the *n* − 1 islands, each with rate *M*_1_/2, leading to the second configuration.
2. One lineage is in the continent and the other in an island. There can be no coalescence event, but three different migration events can occur: if the lineage in the island migrates, which arrives at rate *M*_2_/2, this leads to the first configuration. The lineage in the continent can migrate at rate *M*_1_/2, and it can either reach the island where the other lineage is (leading to configuration 4 below) or migrate to a different island (leading to configuration 3 below).
3. The two lineages are in different islands. No coalescence can occur and any of the two lineages can migrate to the continent, each with rate *M*_2_/2, leading to configuration 2.
4. The two lineages are in the same island. Either a coalescence occurs with rate 1/*c*_2_, leading to configuration 5, or a migration event of one of the two lineages to the continent, each with rate *M*_2_/2, leading to configuration 2.
5. The two lineages have coalesced. This is an absorbing state.

If we replace *M*_2_ by *M* and *M*_1_ by *c*_2_*M*/*c*_1_ in Eq. (3) and normalise population sizes by fixing *c*_1_ = 1, then denoting *c*_2_/*c*_1_ = *c*_2_ = *c* we obtain the following transition rate matrix (see Supplementary Materials for details):

$$Q = \left( {\begin{array}{*{20}{c}} { - 1 - cM(n - 1)} & {cM(n - 1)} & 0 & 0 & 1 \\ {M{\mathrm{/}}2} & { - M(cn - c + 1){\mathrm{/}}2} & {(n - 2)cM{\mathrm{/}}2} & {cM{\mathrm{/}}2} & 0 \\ 0 & M & { - M} & 0 & 0 \\ 0 & M & 0 & { - M - 1{\mathrm{/}}c} & {1{\mathrm{/}}c} \\ 0 & 0 & 0 & 0 & 0 \end{array}} \right).$$

Note that *c* is the ratio between the sizes of the islands and the continent, and that the diagonal entries are obtained by the constraint that the sum over each row is zero.

**Fig. 4:**
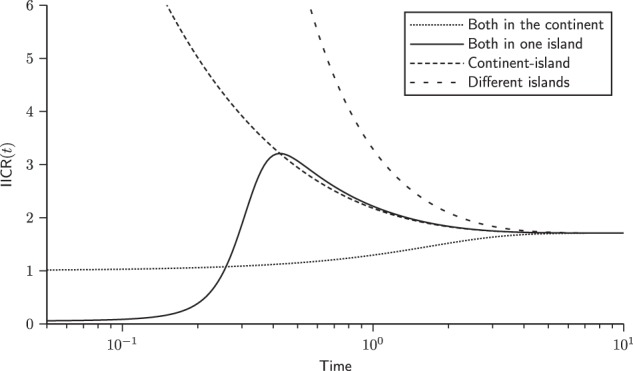
IICR for a continent-island model. We constructed the transition rate matrix for a model with *n* = 4, namely one continent and three same-sized islands. The sizes of the continent and of the islands were set to *c*_1_ = 1 and *c*_2_ = 0.05, respectively. In other words, the continent was 20 times larger than the islands. We set the migration rates to *M*_1_/2 = 0.05, *M*_2_/2 = 1 (note that once *M*_1_ is set, *M*_2_ is constrained to keep inward and outward migrant gene numbers equal, as required by Eq. (1)). In this model there are only four types of IICR curves, two IICR_s_ and two IICR_d_. The first two correspond to the cases where we sample the two lineages either in the continent or in one of the islands. The IICR_d_ curves correspond to cases where one gene comes from the continent and the other from an island or when the two genes come from two different islands

Figure 4 shows the IICR_s_ and IICR_d_ plots for the different sample configurations for a pair of genomes in a continent-island model with *n* = 4 (one continent and three islands). As expected from previous work on the IICR (Mazet et al. 2016; Chikhi et al. 2018), first generation hybrid individuals, whose genome is sampled in different demes, exhibit IICR plots which would be interpreted as expansions from an ancient stationary population, even though the total population size is constant. One of the most striking result is that a diploid individual sampled in one of the islands exhibits an IICR that suggests (forward in time) an ancient stationary population which first expanded before being subjected to a significant population decrease. Thus, different individuals will exhibit very different history, not because their populations were subjected to different demographic histories, but because the IICR does not represent the history of a population. It represents the coalescent history of a particular sample in a particular model.

## The non-stationary structured coalescent (NSSC): constructing the IICR for models with changes in population structure

In this section we extend our work to non-stationary structured (NSS) models under the coalescent and show how the semigroup property can be used to characterise a large family of complex NSS models. The semigroup property allows to compute the probability that a Markov jump process is in a given state at time *t* + Δ*t* by taking into account all its possible states at time *t*. Applied to the structured coalescent, this makes it possible to trace ancestral lineages backward to the MRCA in models where some parameters (*n*, *c*_*i*_, *M*_*ij*_) may change at some time point in the past. In particular, this gives a way to compute (at least numerically) the distribution of coalescence times for a wide family of non-stationary structured models, hence allowing us to introduce and study the NSSC.

### Applying the semigroup property to the structured coalescent

Previous sections showed that to any given stationary structured population model corresponds a transition rate matrix, *Q* that can be constructed and used to predict the IICR for a given sample configuration. Assuming that we sample *k* genes in configuration *α*, we call $T_k^\alpha$ the time to the first coalescence event among these *k* lineages. We also described how the theory of Markov chains allows to compute the probability distribution of $T_k^\alpha$ from *Q* using the formula:

$${\Bbb P}\left( {T_k^\alpha \le t} \right) = P_t\left( {n_\alpha ,n_c} \right) = e^{tQ}\left( {n_\alpha ,n_c} \right),$$

where *n*_*α*_ denotes the index of the configuration *α* and *n*_*c*_ is the number of possible configurations and corresponds to the index of the coalescence configuration.

The matrix *P*_*t*_ (which is the *transition semigroup*) has size *n*_*c*_ × *n*_*c*_ and is obtained by computing the exponential of the matrix *tQ*. The elements of this *n*_*c*_ × *n*_*c*_ matrix are functions of the parameters of the model (*n*, *c*_*i*_, *M*_*ij*_), which are assumed to be constant under the structured coalescent (stationary model). Now, the semigroup property states that for any positive values *t* and *u* we have:

4 $$P_{t + u} = e^{(t + u)Q} = e^{tQ}e^{uQ} = P_tP_u.$$

By using the semigroup property, the structured coalescent can be extended to non-stationary models (*e.g.*, models with changes in the size of one or more demes or in the values of gene flow at some point in the past).

For simplicity, we assume here that the number of demes *n* is fixed for a given species. The reason for doing this is that, once we fix the number of genes sampled at the present (*k*) and the number of demes (*n*), the number of possible states or configurations of the Markov process ($\left| {E_{k,n}} \right|$) is also fixed and so is the size of the corresponding transition rate matrix. It will be thus straightforward to compute products of matrices, using Eq. (4). Keeping *n* constant guarantees that other parameter changes (*i.e.*, *c*_*i*_, *M*_*ij*_) will not modify the state space of the Markov jump process, even if the transition probabilities between these states will change. So, the size of the matrix *P*_*t*_ will always be the same.

Assume that at time *t* = *T* in the past, some of the parameters *M*_*ij*_ or *c*_*i*_ change. This change has no influence on *E*_*k*,*n*_ and does not affect the evolution of the process between *t* = 0 and *t* = *T*. Denote by *Q*_0_ the transition rate matrix of the Markov chain for 0 ≤ *t* ≤ *T* and *Q*_1_ the corresponding transition rate matrix for *t* > *T*. If we call $\tilde P_t$ the *transition semigroup* of the Markov chain that models this structured scenario with a demographic change event at time *T*, we can compute $\tilde P_t$ by using the semigroup property as follows:

$$\tilde P_t = \left\{ {\begin{array}{*{20}{l}} {e^{tQ_0},} \hfill & {{\mathrm{if}}\,t \le T} \hfill \\ {e^{TQ_0}e^{(t - T)Q_1},} \hfill & {{\mathrm{otherwise}}.} \hfill \end{array}} \right.$$

In particular, the distribution of $T_k^\alpha$, the first coalescence time of *k* genes sampled in configuration *α* under this structured model with a past demographic change event, can be computed by:

$${\Bbb P}\left( {T_k^\alpha \le t} \right) = \tilde P_t\left( {n_\alpha ,n_c} \right)$$

The *pdf* of $T_k^\alpha$ can then be computed by $f_{T_k^\alpha }(t) = \tilde P_t^\prime \left( {n_\alpha ,n_c} \right),$ where

$$\tilde P_t^\prime = \left\{ {\begin{array}{*{20}{l}} {e^{tQ_0}Q_0,} \hfill & {{\mathrm{if}}\,t < T} \hfill \\ {e^{TQ_0}e^{(t - T)Q_1}Q_1,\;\;} \hfill & {{\mathrm{otherwise}}.} \hfill \end{array}} \right.$$

This procedure can be extended to any number of parameter changes, by defining the respective transition rate matrices for each of the time intervals between successive changes in the parameters of the structured model. Thus, the distribution of coalescence times (and the IICR) for structured models in which migration rates and demes sizes can arbitrarily change, can be obtained from the computation of matrix exponentials and matrix products.

Moreover, the NSSC framework allows to compute the IICR for models considering a population split. For example, a model considering one ancestral population that separated into two subpopulation at time *T* can be easily approximated under the NSSC framework. To do this, just set a value of gene flow from the present to time *T*. Then set a gene flow equal to infinity (in practice we use a gene flow high enough so that the two populations behave as a panmictic one) from time *T* to the past. The following section considers a more general model of population split that gives a new perspective to the history of evolution of humans and Neanderthals.

### Application: humans and Neanderthals IICR

**Fig. 5:**
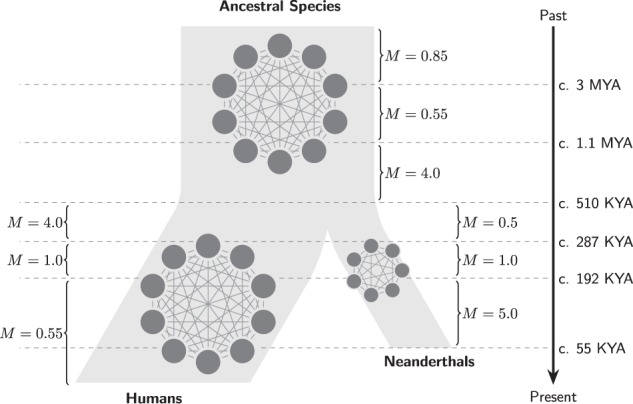
Hypothetical scenario presenting humans and Neanderthals as structured species derived from an unknown *Homo* species that was itself structured. The times at which gene flow (*M*) changed are indicated by horizontal lines

**Fig. 6:**
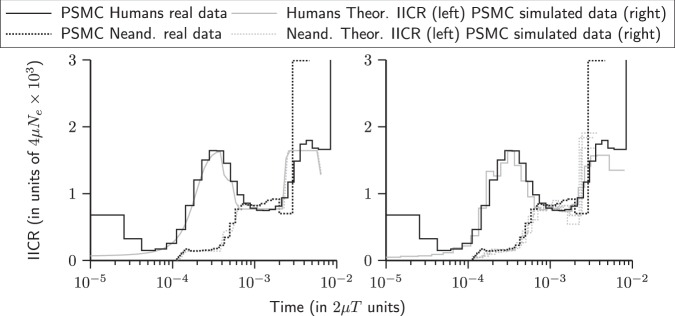
IICR and PSMC plots for humans and Neanderthals. The PSMC plots obtained from real human and Neanderthal sequences are similar to the theoretical IICR (left panel) corresponding to the proposed scenario. Also, they are similar to the PSMC plots obtained from sequence data simulated under the proposed scenario (right panel)

In this section we show how the NSSC can be used to identify a single model (Fig. 5) incorporating both humans and Neanderthals as structured species derived from an unknown ancestral *Homo* species that was itself structured. This is possible because of the properties of the IICR in structured model as described and discussed in Mazet et al. (2016) and Chikhi et al. (2018). For instance, these authors have shown that the IICR (and thus the PSMC plots) are constrained by a set of simple rules which we used to construct PSMC plots identical to those inferred from human and Neanderthal genomes (see details below). For instance, the previous IICR studies have shown that increasing (resp. decreasing) the migration rate M will move the IICR downward (resp. upward). Changing the timing of several of such changes will create humps that will shift in time. Also, the recent level of the IICR will be influenced by the sampling scheme and the local deme size. Finally, low and high M values will impact the speed at which the IICR moves upward as time goes backward. Based on these and other results obtained by Chikhi et al. (2018), and on the model proposed by Mazet et al. (2016) we were able to rapidly construct a model that predicted the IICR for humans and Neanderthals in a single model using the NSSC framework. This model assumed that one diploid was sampled in a human deme and another in a Neanderthal deme. We then used several validation steps. Following the approach used by Chikhi et al. (2018) we also computed the IICR using *T*_2_ values simulated with Hudson’s *ms* software for the same demographic scenario. The PSMC curves inferred from real data were then plotted for comparison (Fig. 6). As an additional validation step we also plotted (Fig. 6 right panel) the PSMC inferred from genomic data simulated with *ms* (*i.e.*, DNA sequences rather the *T*_2_ values) for the same scenario together with the PSMC from the real sequences. The proposed scenario was constructed to explain the PSMC plots observed in humans and Neanderthals, and not to explain other statistics computed from genomic data. For instance, we used *n*-island models which are symmetrical models. This means that our model cannot separate one geographical region from another. It is important because several statistics have been computed that indicate some asymmetry between geographical regions. Our model cannot and does not aim to explain such statistics. To do that, and as already stressed by Mazet et al. (2016) and Chikhi et al. (2018), spatial structure will be necessary, as well as some form of asymmetry. This is not the aim of this simple example. The Neanderthal case would require a proper and full independent study with a model that would explain all statistics. We also stress that current structured models cannot explain the PSMC plots.

In the proposed and “manually inferred” scenario (see Fig. 5), humans and Neanderthals descend from a *Homo* species that was structured in ten interconnected demes, as in Mazet et al. (2016), and whose connectivity changed around 3 million years ago (MYA) when the migration rate *M* = 4 *Nm* decreased from 0.85 to 0.55. Then, around 1.1 MYA, *M* increased significantly from 0.55 to 4. The following period of reasonably high connectivity (*M* = 4 corresponds to an *F*_st_ of 0.11 across the whole species) was maintained in the lineage that led to humans until 0.287 MYA whereas a significant change occurred when Neanderthals split from that common lineage, some time about 0.51 MYA. Our model suggests that to fit the estimated Neanderthal PSMC results the original Neanderthals are the result of a “sub-sampling” or split from human demes (*n* = 7 demes in our model). These new Neanderthal demes were around 16% of the size of human demes. At the same time (0.51 MYA) *M* decreased from 4 to 0.5 in the Neanderthal lineage whereas, as noted above, it remained constant in humans. In the case of Neanderthals, the reduction is surprisingly close to the level of connectivity of the ancestral species (between 3 and 1.1 MYA). It is as if archaic Neanderthals were a group of small demes that derived from human demes and that had gone back to an ancestral low connectivity state. Neanderthals stayed in that low connectivity state until 287 KYA. One striking result is that a simultaneous change is observed at that time in humans and Neanderthals, and that it is now in the opposite direction. Whereas gene flow started to decrease in humans, from *M* = 4 to *M* = 1, it doubles in Neanderthals from *M* = 0.5 to *M* = 1. Then, around 192 KYA, gene flow increases to *M* = 5 in Neanderthals and decreases to *M* = 0.55 in humans. It is as if in a period of 100 KY Neanderthals’ gene flow had increased 10-fold, perhaps as a consequence of a geographic contraction. Humans on the other hand appear to have maintained a low connectivity until the Neolithic as discussed in Mazet et al. (2016). Assuming a mutation rate per generation equal to 1.25 × 10^−8^, the proposed scenario is consistent with a deme size of 1276 for humans and a deme size of 200 for Neanderthals. Note that under this scenario, deme sizes remain constant and the PSMC patterns can be explained only by changes in connectivity. Note also that in this figure, we did not simulate the Neolithic expansion, which is why the human IICR and PSMC plots continue to decrease to the local deme size in the recent past, as explained in Mazet et al. (2016) and Chikhi et al. (2018).

If we trace the theoretical IICR corresponding to the scenario described above, we can see that it is similar to the PSMC plots obtained from real human and Neanderthal data (Fig. 6, left panel). Moreover, we simulated 40 full genome length (*i.e.*, 3 GB) sequences with *ms* under the proposed scenario. The first 20 corresponded to a genome sampled in a human deme and the last 20 corresponded to a genome sampled in a Neanderthal deme. We then applied the PSMC to each of these simulated sequences and compared the results with the PSMC plots obtained from real data (Fig. 6, right panel).

The absolute dates presented here should be taken with a grain of salt since they depend on various parameters which we took from previous studies. In Mazet et al. (2016) and Chikhi et al. (2018) we used the mutation rates of Li and Durbin (2011) but here we used the values of Prufer et al. (2014) to be able to compare our IICR results to the PSMC results obtained by the latter study.

Altogether, these results show that the scenario proposed explains the skyline plots obtained by PSMC from real data. It is thus possible to construct a scenario in which humans and Neanderthals are structured and descend from a common ancestral species that was also structured. PSMC plots are usually interpreted in terms of population size change. However, this scenario explains PSMC plots without any change in population size in humans, and with a split, disconnection and deme size reduction in Neanderthals. The scenario, however, requires neither gene flow nor admixture between humans and Neanderthals. The simple fact of sampling diploids in different demes (humans or Neanderthals) generates the very different PSMC plots inferred for humans and Neanderthals.

## Discussion and perspectives

### The NSSC as an extension of the structured coalescent

The theoretical framework presented in this study is closely related to Herbots’ works (Herbots 1994; Wilkinson-Herbots 1998), who introduced the use of transition rate matrices for studying structured models and computed the coefficients of the transition rate matrix for many stationary models. Here we extended the existing theory to non-stationary structured models (see also Hobolth et al. (2011)). This can impact future population genetic studies in several important ways. The NSSC framework gives a theoretical way for computing the *cdf* and the *pdf* of *T*_2_ under a wide family of models of structured population. It also includes a natural way of incorporating past demographic events (*i.e.*, changes in deme sizes and/or in gene flow) into models of population structured. Currently, most of the population genetic studies either assume panmixia and try to infer past changes in population size or consider structure but then often assume a rather constrained tree structure in which each branch is assumed to be panmictic even if it may correspond to a species or a continental population. The parameters inferred under such models may be very relevant if the model is robust or misleading if it is not. At this stage it is unclear how models used to study human evolution may actually be as robust as they are assumed to be. The NSSC framework developed here is original because it allows to combine changes in population structure and size into the same model. Allowing to incorporate past demographic events into a model considering population structure is a step forward that may help to disentangle the confounding effects of structure on methods used to reconstruct demographic history that has been pointed by previous studies (Wakeley 1999; Storz and Beaumont 2002; Städler et al. 2009; Chikhi et al. 2010; Heller et al. 2013).

The NSSC allows us to compute IICR curves with much lower computational cost than the simulation based approach used in Chikhi et al. (2018). This gives the possibility to test alternative scenarios and also lays the theoretical bases to implement an inferential framework using the IICR computed from genomic data by methods like the PSMC (Li and Durbin 2011) or MSMC (Schiffels and Durbin 2013). However, whereas inference can be done by hand as explained above, the development of an automated inferential process as well as the corresponding validations for simple and complex models would need a full and independent study. It would be necessary to define a distance measure between the simulated IICR and observed PSMC plots, and find efficient algorithms to explore the parameter space of structured models. This is an ambitious goal that the NSSC should help reach.

We would also like to stress that the hypothesis given in (1) was originally given by Herbots (1994) as necessary for convergence and was followed throughout the manuscript for consistency. However, it may not be as crucial as it originally seemed. It appears that it can be removed without affecting the convergence to the continuous-time Markov process (Kozakai et al. 2016). This makes it possible to apply the Markovian approach used here to more general models than the ones discussed in this work. We also note that several studies based on models ignoring condition (1) have already been published (Notohara 1990; Costa and Wilkinson-Herbots 2017). This also includes our own work where we used *ms* simulations (Hudson 2002) because this software does not require this assumption to work (Chikhi et al. 2018).

### Humans, Neanderthals, and genomic story-telling

Story-telling seems to be pervasive in human evolutionary biology, probably because of the limited amount of available data and the difficulty to separate alternative models. While genetic data should provide a relatively objective source of information (since we know how genetic data are transmitted across generations) the models used by geneticists to fit parameters can profoundly change our understanding of our past. Since it is not always clear how a structured model should objectively be chosen while being also computationally tractable, this model choice step leaves some space for story-telling, which we could not avoid either. While the scenario proposed here (in Fig. 5) should not be taken at face value, it is important to see that it profoundly differs in its conclusions from current models, including structured models. One major difference is that the timings of events in a panmictic or structured model can be quite disconnected at the level of the IICR. The reason for this is that the IICR has its own dynamics even under stationarity. This produces apparent increases or decreases in population size which have no actual associated demographic event (Mazet et al. 2016; Chikhi et al. 2018). Under a panmictic model any change in the IICR must be due to some causal event that makes the IICR go up or down.

In our scenario we found that one major event dated around 300 KYA induced a change in connectivity that was simultaneous in humans and Neanderthals. If our model captures something meaningful, this would suggest a striking consistency across the two species. One interpretation could be that the two *Homo* species responded to the some environmental change, around 300 KYA. This could be tested. Our model differs also from current models by the fact that it explains the decrease in the Neanderthal PSMC plots, not as a decrease in population size but rather as a result of two different forces. One is a decreased isolation of Neanderthal populations, and the second a consequence of the properties of the IICR in structured models. Indeed, the “humps and bumps” of IICR plots (Chikhi et al. 2018) can be caused by changes in connectivity or by a constitutive property of the IICR (Mazet et al. 2016; Chikhi et al. 2018). It would be important to determine if there are data suggesting a reduction and increased connectivity of Neanderthals. Finally, in our model, when Neanderthals split from the common ancestral species, they have much smaller demes than humans and these demes are less connected. It is interesting to note that a recent genomic study by Rogers et al. (2017) suggested that Neanderthals were probably distributed in small and isolated demes. Our results are thus consistent with that idea.

In a recent study Kuhlwilm et al. (2016) used a complex model with splitting populations to represent the evolution of humans, Neanderthals and Denisovans. Their model was not inferred from the data but rather chosen *a priori* and probably on the basis of beliefs (or knowledge) that the authors had gathered. While they did carry out several validation steps, the model was not inferred from the data. Based on our understanding of the IICR in structured models (Mazet et al. 2016; Chikhi et al. 2018), it seems very unlikely that their model could explain the PSMC curves of humans and Neanderthals. For instance their model assumes constant population sizes for long periods and ignores gene flow. In order to produce the humps observed in humans and the decrease of Neanderthals PSMC plots, one at least of the two is typically necessary (Mazet et al. 2016; Chikhi et al. 2018). The admixture events that are incorporated in that model will likely produce a large hump, but it is unclear why it would not be visible in Neanderthals then, if there was indeed gene flow towards them as has been claimed. It would thus be important to see how adding gene flow in their model would change admixture estimation while producing the right PSMC plot shapes. It is very possible that admixture would still be necessary to explain some patterns of genomic diversity observed in humans.

Our model has also many limitations. For instance, it ignores spatial structure even though we have noted in a previous study (Chikhi et al. 2018) that to understand human evolution, spatial models such as stepping stone models will be necessary to explain the variability observed in human PSMC plots. For Neanderthals similar claims cannot be made yet since only one Neanderthal PSMC plot has been published to date. Another issue with our model is that by using *n*-island models within each species, we implicitly assume that all human populations are statistically exchangeable. Such a model cannot thus explain various statistics that have been found to vary between human populations, including the D statistic used to argue for Neanderthal admixture. We should also note that our model assumes that population size changes are negligible. This does not mean that we believe that there were no size changes in the history of humans or Neanderthals. Allowing for deme size changes is easy under the NSSC framework, but it would likely make the inferential process much more complex.

At this stage, one should be very careful regarding the interpretation of Neanderthal and human genomic data. Story-telling is easy and we need a well-defined statistical framework to better explore the extremely large parameter space of structured models. While the presented scenario does not aim to explain all the complexity of human and Neanderthal evolution it explains genomic patterns (PSMC plots) that are currently not explained by existing admixture models. We thus conclude with Chikhi et al. (2018) and Eriksson and Manica (2012) that claims of admixture may be weaker than usually believed, even if we must also conclude that admixture cannot be excluded today.

Beyond humans and Neanderthals, the NSSC framework presented here should now be developed as a full automated inferential tool to identify quickly and efficiently models that can, and models that cannot, explain known genomic features. Changes in connectivity in a complex splitting model produce complex genomic patterns that cannot be easily interpreted. By using the IICR and the NSSC we were able to re-interpret human and Neanderthal evolution, while stressing that it is only one of probably many possible interpretations. We mainly used models without changes in population size but we do not believe that there were no such changes in the history of most species. It however means that such changes are not always necessary to explain the data and that changes in connectivity should be better integrated in our understanding of the recent evolution of species (Chikhi et al. 2010; Mazet et al. 2016; Chikhi et al. 2018). Mazet et al. (2016) and Chikhi et al. (2018) showed how different individuals from the same species can exhibit very different “demographic histories” simply because they or their genes were sampled in different locations of a structured population. The transition rate matrices approach can make the computation of the IICR extremely efficient. This suggests that the IICR can be computed for various models and compared to observed PSMC plots. It can thus be used as a summary of genomic data and estimated with the PSMC and MSMC methods, as suggested by Chikhi et al. (2018) to exclude models or identify the best models.

### Increasing the sample size to more than two sequences

The Markov process approach used in sections ‘The structured coalescent and transition rate matrices: towards the IICR’ and ‘The non-stationary structured coalescent (NSSC): constructing the IICR for models with changes in population’ allows to trace back ancestral lineages coming from a sample of arbitrary size. This means that we can compute the distribution of the first coalescence event in a sample of *k* genes (denoted *T*_*k*_) for *k* ≥ 2. Thus, it is theoretically possible under the NSSC framework to obtain statistical properties of the underlying genealogical tree for samples of size *k*. However, in this study we mainly focused on the IICR as defined by Mazet et al. (2016) for *T*_2_. The reason for this is that when *k* ≥ 3 the number of states to consider in the Markov process becomes very large and so does the corresponding transition rate matrix. It becomes messy to enumerate all the states and to construct the corresponding transition rate matrix. Moreover, the computation of the matrix exponential becomes intractable under the classic numerical methods (Moler and Loan 2003). Some optimisations need to be done taking advantage of the particular structure of the matrices associated to the NSSC framework. Also there is a need for a clear algorithm enumerating all the possible states when tracing back more than two ancestral lineages to the MRCA. It may also be possible to construct a ‘reduced’ transition rate matrix instead of the full one if there are ‘symmetries’ in the model. For instance, the *n*-island model is highly symmetrical (all islands have the same size and migration rates are identical between all islands). The advantage of using symmetries is that it significantly reduces the size of the transition rate matrix and computation time but this idea will not be viable for all structured models.

In conclusion, one of the great challenges of population genetics inference is to identify the structured models that could explain existing genomic data. Until now the choices of structured models has been to a large extent arbitrary. The NSSC modelling framework proposed here may be a powerful and promising way to overcome that challenge, and perhaps reduce arbitrariness and some level of story-telling that has often plagued human evolution discourse. All scripts used to carry out the simulations and analyses for this work are available at: https://github.com/willyrv/nssc-tools.

## Electronic supplementary material

The IICR and the non-stationary structured coalescent: towards demographic inference with arbitrary changes in population structure
