# Supplementary material for: The IICR and the non-stationary structured coalescent: towards demographic inference with arbitrary changes in population structure
Source: Heredity (Edinb). 2018 Oct 7;121(6):663–78. doi: 10.1038/s41437-018-0148-0 (PMC6221895; doi:10.1038/s41437-018-0148-0)
Supplement: Supplementary file 1 — The IICR and the non-stationary structured coalescent: towards demographic inference with arbitrary changes in population structure [file 41437_2018_148_MOESM1_ESM.pdf]

Supplementary Information for

The IICR and the non-stationary structured coalescent: towards demographic inference with arbitrary changes in population structure

Willy Rodríguez, Olivier Mazet, Simona Grusea,
Armando Arredondo, Josué M. Corujo, Simon Boitard and Lounès Chikhi

Corresponding author: Lounès Chikhi
Email: `lounes.chikhi@univ-tlse3.fr`

This PDF file includes:

Supplementary text

Figures S1 to S10

1 General algorithm for the construction of the transition rate matrix for two lineages

We give a general algorithm that can be used to construct the transition rate matrix of a given model. The first step is to explicitly order all the demes. Then, given the number n of (ordered) demes the set of all possible configuration for $k = 2$ lineages is:

$$E_{2,n} = \{\alpha \in \mathbb{N}^2, \alpha = \epsilon^i + \epsilon^j \text{ with } i, j = 1, \ldots, n\} \cup \{c\},$$

where $\epsilon^i + \epsilon^j$ means that there is one lineage in deme i and one lineage in deme j (note that it could be $i = j$); and c is the configuration where both lineages have coalesced.

We take the inverse lexicographical order on $E_{2,n}$. Define ϕ as a function from $E_{2,n}$ to $\{1, 2, ..., |E_{2,n}|\}$ such that $\phi(\alpha)$ is the index of α according to the inverse lexicographical order. Then ϕ^{-1} is the inverse of ϕ and $\phi^{-1}(i)$ gives the element of $E_{2,n}$ which is at position i according the inverse lexicographical order.

Once the function ϕ is defined and we have the values of $C = (c_1, \ldots, c_n)$ (the size of the demes) and M_{ij} (the migration matrix), we can use the following algorithm to construct the transition rate matrix Q:

```
procedure CREATEQMATRIX(C, M)                     ▷ (C: deme sizes; M: migration matrix)
    n ← length(C)                                 ▷ Initialisation; number of demes
    n_c ← n(n + 1)/2 + 1                          ▷ Initialisation; number of states
    Q ← n_c × n_c matrix full of zeros            ▷ Initialisation; transition rate matrix
    for k in {1 . . . n_c − 1} do
        (x_1, x_2, . . . , x_n) ← φ^{-1}(k)
        for i in {1 . . . n} do
            if x_i > 0 then
                for j in {1 . . . n} do
                    if j ≠ i then
                        (y_1, y_2, . . . , y_n) ← (x_1, x_2, . . . , x_n)    ▷ migration events
                        y_i ← x_i − 1
                        y_j ← x_j + 1
                        l ← φ(y_1, y_2, . . . , y_n)
                        Q_{k,l} ← x_i M_{i,j}
                    end if
                end for
                if x_i = 2 then
                    Q_{k,n_c} ← 1/c_i                 ▷ coalescence events
                end if
            end if
        end for
    end for
    for k in {1 . . . n_c − 1} do
        Q_{k,k} ← − Σ_{l≠k} Q_{k,l}               ▷ rows of the matrix Q must sum to zero
    end for
    return Q
end procedure
```

Note that since the last configuration (coalescence) is an absorbing state of the Markov process, the last row has only zeros.

2 Constructing the IICR for stationary models. Examples: stepping stone and continent-island

We now apply the framework and algorithm described above to some stationary models. By a stationary model we understand a structured model in which the parameters (i.e., number of demes, sizes of demes and gene flow) remain constant over time. To our knowledge, there is no analytical expression for the distribution of the coalescence time T_2 under most of these models. For some of them it is possible to find a simplified transition rate matrix using some symmetries. In those case we give the corresponding transition rate matrix Q that can be used to compute numerically the distribution of T_2 and the IICR. In other cases it is not possible to get a simplified version of Q and we used the algorithm given in section 1 to obtain the IICR.

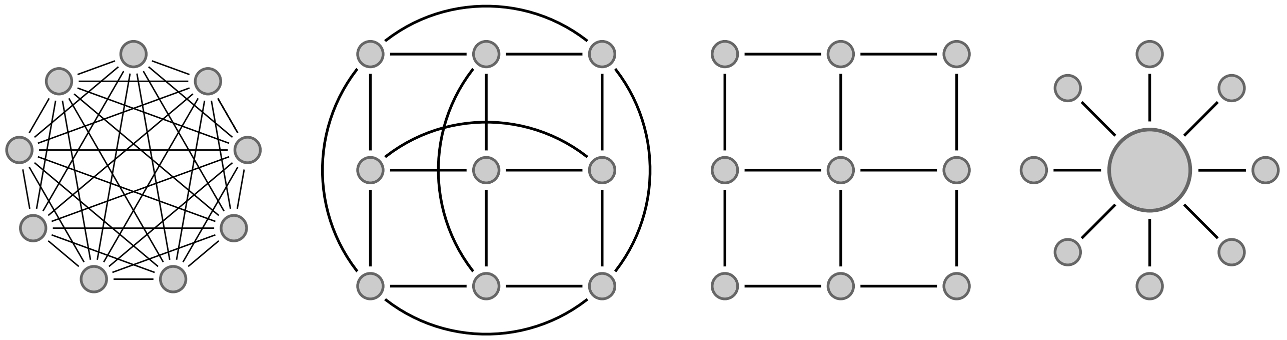

Figure S1: Diagrams for commonly used structured models. From left to right: n-islands, torus 2D stepping stone, 2D stepping stone and continent-island model.

2.1 stepping-stone models

Stepping stone models (Kimura, 1953; Malécot, 1948) assume that the demes are located at the nodes of a regular lattice in one or two dimensions (hereafter 1D and 2D stepping stone models). Each deme can have up to four neighbours and migration events are only possible between neighbouring demes. These models incorporate space, and are thus thought to be more realistic than the n-island model described above, which implicitly assumes that migration is as likely between neighbours as it is between distant islands. The border demes can either be connected with each other, hence forming a torus, or can behave as bouncing borders (Figure S1). In some models the bouncing borders migrants are assumed to stay in their deme, whereas in other models they are distributed among the demes to which their deme is connected.

We will distinguish two cases:

1. Without edges: One dimension (1D circular stepping stone) and two dimensions (2D torus stepping stone). They are more symmetric since all the migration rates are equal.

2. With edges: 1D and 2D stepping stone. Islands located on the edges and in the corners have fewer neighbours than islands in the middle of the lattice. In order to maintain simplicity and symmetry, the same migration rate is taken between each pair of islands. This implicitly assumes that migrants trying to migrate "outside" are bouncing back to their deme of origin. As we will see there are still more parameters in the model, and the corresponding transition rate matrices are more complex.

We will give an example of each of the four combinations: one or two dimensions, and with and without edge effects.

2.1.1 Circular 1D stepping-stone model

Here we assume that the population is divided into n ($n \geq 2$) equal-sized islands which are located on a circle (Figure S2). Each island thus receives immigrants coming only from its two neighbours.

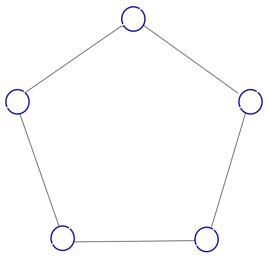

Figure S2: 1D circular stepping stone with 5 islands

With the notations of the main manuscript, $\forall i = 1 \dots n$ we set $c_i = 1$, $M_i = M$, and $M_{ij} = M/2$ if $|i-j| = 1$ or $|i-j| = n-1$, $M_{ij} = 0$ if not.

The symmetry of the model allows us to consider that the configuration of a sample of two lineages depends only on their distance d, defined as the number of islands separating them, d ranges from 0 to $\lfloor n/2 \rfloor$ ($\lfloor x \rfloor$ is the largest integer not larger than x), that is, $\lfloor n/2 \rfloor + 1$ different values.

The corresponding matrix Q is then of size $\lfloor n/2 \rfloor + 2$, the last configuration corresponding to the coalescence event, which can occur only if both lineages are in the same island. When there are five demes ($n = 5$), then we have $\lfloor n/2 \rfloor = 2$, the simplified transition rate matrix Q has thus 4 rows and columns:

$$Q = \begin{pmatrix} -1-2M & 2M & 0 & 1 \\ M & -2M & M & 0 \\ 0 & M & -M & 0 \\ 0 & 0 & 0 & 0 \end{pmatrix}.$$

The first row represents the transitions away from the configuration in which both lineages are in the same island. They coalesce with rate $1/c_i = 1$. Each lineage can migrate with rate $M/2$ towards any of the two neighbouring islands. Any of these migrations will lead to a configuration in which both lineages are in a pair of islands distant of 1 unit (this is the second configuration that we consider).

From this second configuration (corresponding to the second row), no coalescence can occur and each lineage can only migrate to the next island, leading to two possible configurations. Either the migration brings them back on the same island (and we are back to the first configuration with rate $M/2$) or one of them migrates to the next island hence increasing the distance between them by one unit to 2 units (this is the third configuration). Since there are $n = 5$ islands there cannot be a distance greater than two (islands 2 and 5 or islands 1 and 4 are only 2 units distant) and we have thus all possible configurations of the simplified matrix Q. Also, since $n = 5$ is odd, migration events from this third configuration can only lead to configurations that are identical to itself or to the second one (with rate $M/2$) (see Figure S2). Some IICR corresponding to the circular stepping stone are shown in Figures S3, S4 and S5.

When there are six demes ($n = 6$), then we have $\lfloor n/2 \rfloor = 3$, the simplified matrix Q has thus 5 rows and columns:

$$Q = \begin{pmatrix} -1-2M & 2M & 0 & 0 & 1 \\ M & -2M & M & 0 & 0 \\ 0 & M & -2M & M & 0 \\ 0 & 0 & 2M & -2M & 0 \\ 0 & 0 & 0 & 0 & 0 \end{pmatrix}.$$

The only difference with the previous example is the fourth configuration, which corresponds to the largest distance of 3 units. From that configuration all migration events necessary lead to the third configuration (corresponding to a distance of 2).

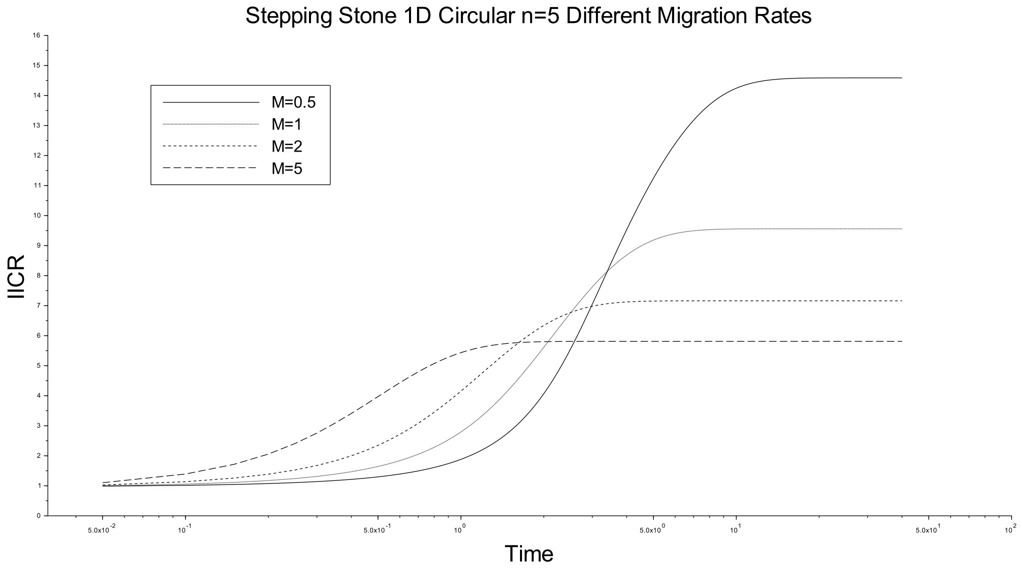

Figure S3: 1D circular stepping stone, $n = 5$, different values of M

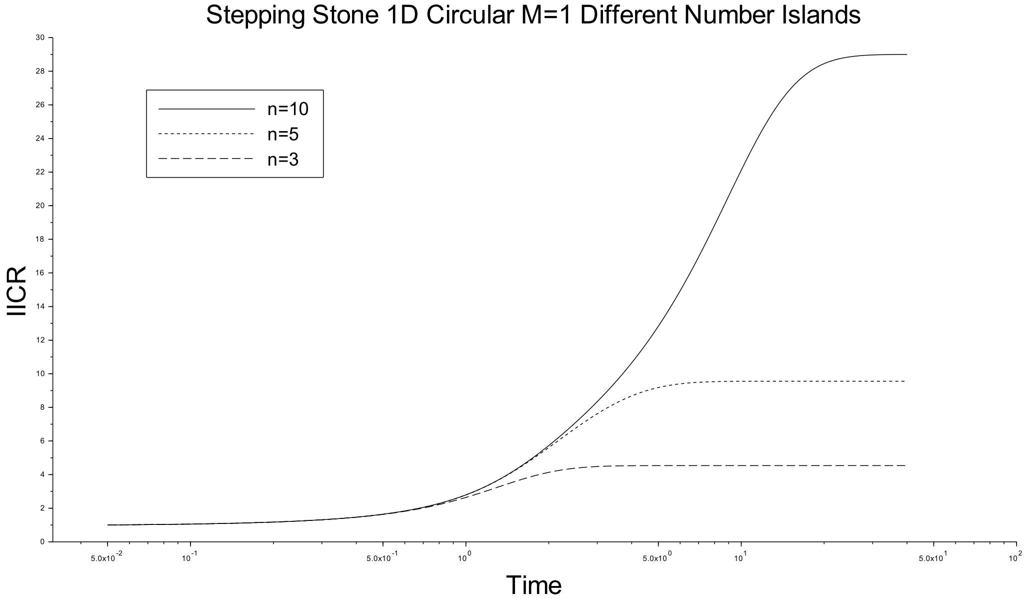

Figure S4: 1D circular stepping stone, $M = 1$, different values of n

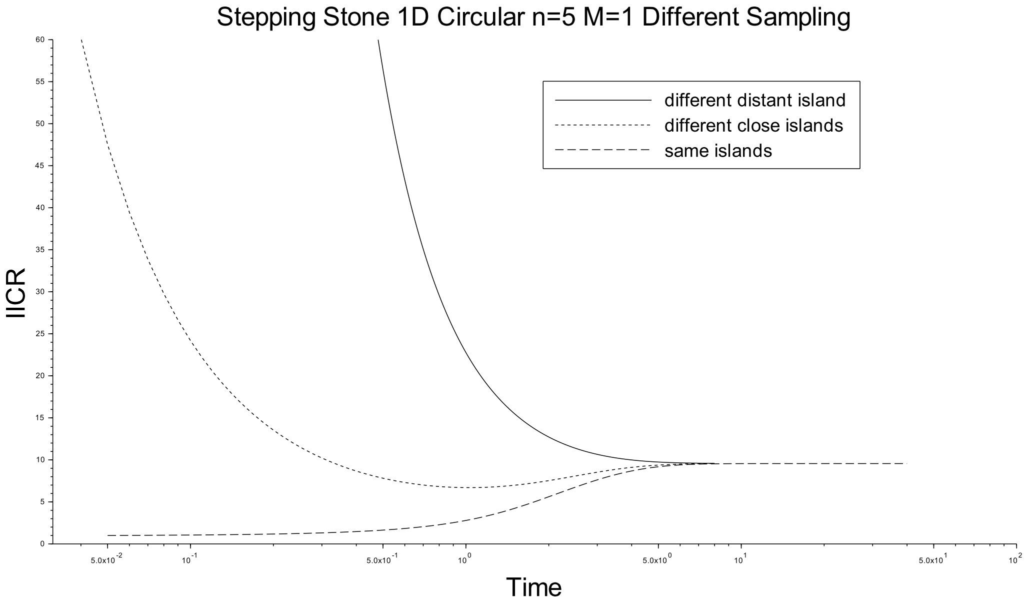

Figure S5: circular stepping stone, $n = 5$, $M = 1$, different sampling : two lineages in the same island, two lineages in nearby islands, two lineages in distant islands

2.1.2 1D stepping-stone model with bouncing edges

Here we consider the edge effects since the two islands located at the extremes of the 1D stepping stone have only one neighbour. The population is divided into n ($n \geq 2$) equal-sized island (see Figure S6).

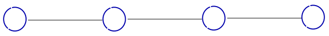

Figure S6: 1D stepping stone with 4 islands

Keeping the same notations, $\forall i = 1 \dots n$ we set $c_i = 1$, and $M_{ij} = \frac{M}{2}$ if $|i - j| = 1$, $M_{ij} = 0$ if not.

Since there are fewer symmetries than in the circular model, there are significantly more possible configurations in the simplified transition rate matrix Q and we now have to take into account the distance between the two lineages, and the distance from the edge of the linear stepping stone.

The general case can be analysed using combinatorics approaches but this will not be presented here and we will simply give the results for $n = 4$. Even in this case the simplified version of the transition rate matrix Q has as many as seven rows and seven columns. If we denote by (i, j) the configuration when one lineage is in island i and the other in island j, with $i, j = 1 \dots 4$, and given the central symmetry of the model, we can enumerate the configurations as follows :

1. $(1, 1)$ which is the same as $(4, 4)$
2. $(1, 2)$ which is the same as $(3, 4)$
3. $(1, 3)$ which is the same as $(2, 4)$
4. $(1, 4)$
5. $(2, 2)$ which is the same as $(3, 3)$

6. $(2,3)$
7. coalescence c

This allows us to construct the corresponding matrix Q:

$$Q = \begin{pmatrix} -1-M & M & 0 & 0 & 0 & 0 & 1 \\ M/2 & -3M/2 & M/2 & 0 & M/2 & 0 & 0 \\ 0 & M/2 & -3M/2 & M/2 & 0 & M/2 & 0 \\ 0 & 0 & M & -M & 0 & 0 & 0 \\ 0 & M & 0 & 0 & -1-2M & M & 1 \\ 0 & 0 & M & 0 & M & -2M & 0 \\ 0 & 0 & 0 & 0 & 0 & 0 & 0 \end{pmatrix}.$$

The IICR corresponding to a 1D stepping stone with bouncing edges is shown in Figure S7.

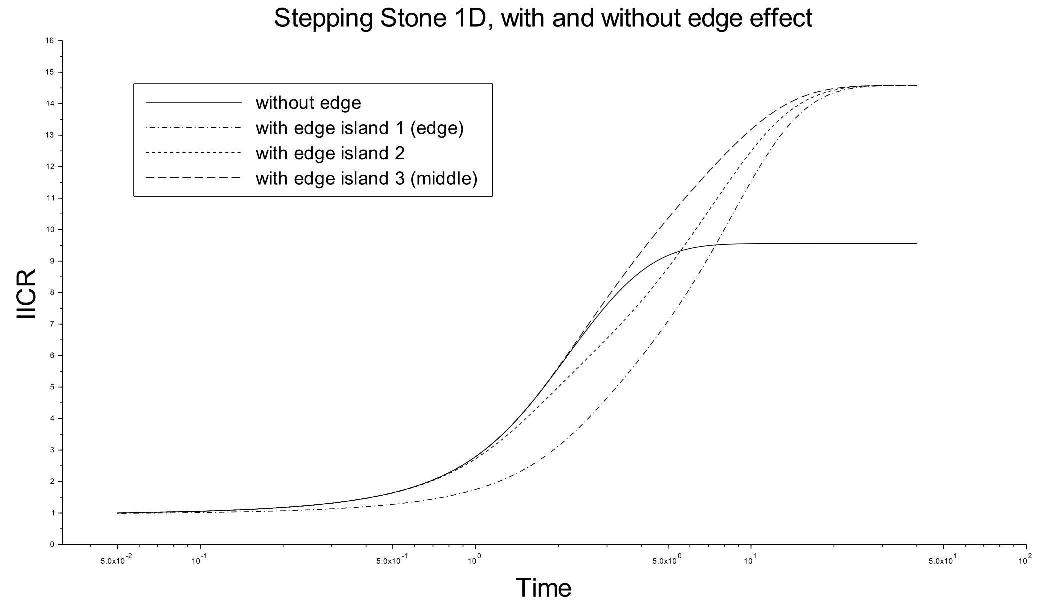

Figure S7: Comparison of two 1D Stepping Stone Models: with and without edge. Number of demes $n = 5$ and gene flow $M = 1$. Sampling two lineages in the same deme. When there is edge effect, we present the three ways to sample in the same island: extreme deme (number 1 or 5), demes right next to the extreme (2 or 4) and the middle one (number 3).

2.1.3 2D stepping stone models with and without edges

For the 2D stepping stone model, we set, $\forall i, j = 1, \dots, n$, $c_i = 1$ and $M_{ij} = M/4$ if islands i and j are neighbours, and $M_{ij} = 0$ otherwise. The difference between the models with and without edges used here is thus in the way neighbours are defined. In the model with borders the four corner islands have only two neighbours, the islands on the edges of the lattice have three, and the others have four neighbours (see Figure S1). In the 2D stepping stone model, we computed the corresponding transition rate matrix from the migration matrix of the model using the algorithm given in section 1.

Figure S8 shows the IICR_s (two haploid genomes sampled in the same deme, or one diploid genome), for a 3×3 stepping stone model with and without borders (Figure S1). In the latter case (no borders), all demes are statistically identical, and there can thus be only one IICR_s

plot. In the model with borders, there are three possible ways to sample a diploid individual, and three IICR_s are plotted. This figure confirms the results of Chikhi et al. (2018) by showing that the IICR_s plots for a stepping stone are also S-shaped. They all start in the recent past at a value equal to the deme size and converge in the ancient past towards the same plateau. However, it is remarkable that they differ in the trajectory from the present to the plateau value, depending on the location of the deme (corner, border or centre). These results thus confirm that in a stepping stone model, two diploid individuals sampled in different demes (i.e., geographical regions) will both exhibit signals of population decrease that will be different even though the population size was constant and they both belonged to the same structured model (Chikhi et al., 2018).

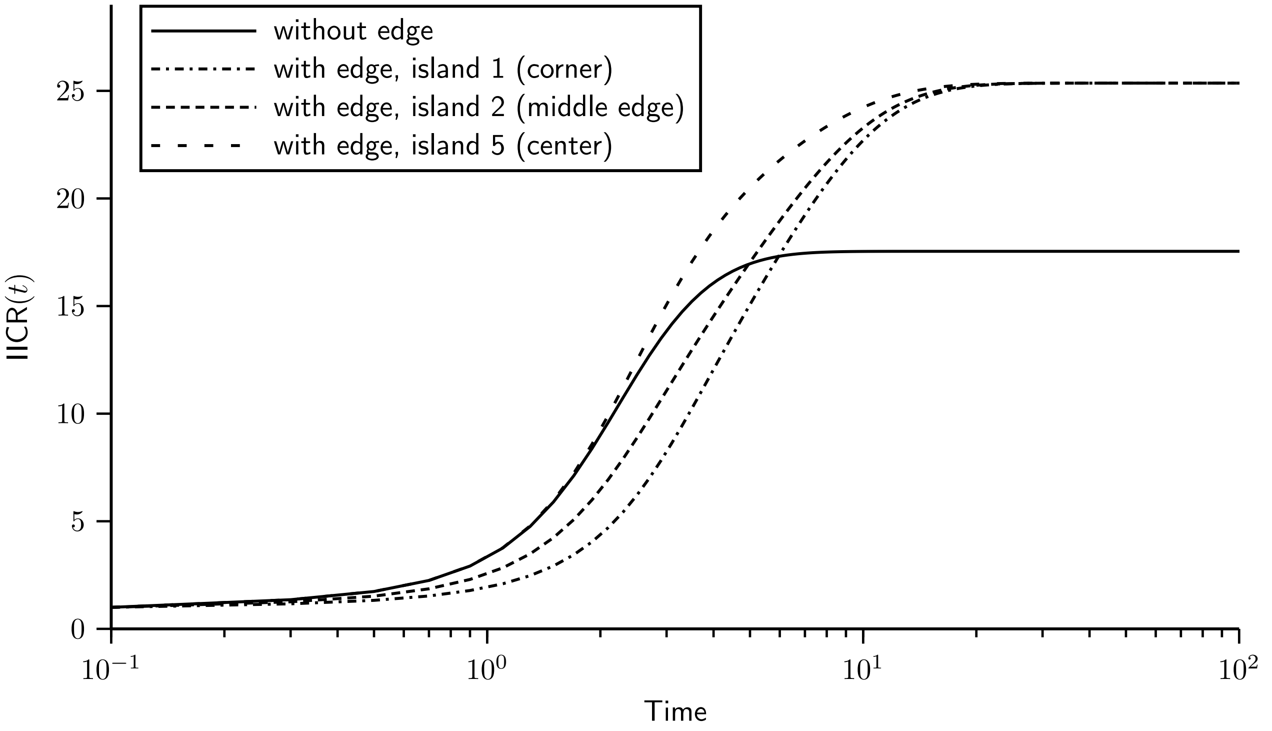

Figure S8: IICR plots for the 2D stepping stone model. Here we assumed a model with $3\times3 = 9$ islands and $M = 1$, with and without edge effect. In the model with edge effect, we plot the three ways to sample two lineages in the same island: in island 1, 3, 7 or 9 (corner), in island 2, 4, 6 or 8 (middle of the edge), and in island 5 (center of the lattice).

2.2 Continent-island model

2.2.1 General case

Here we assume a model where the population is divided into n demes (one big deme called *continent* and $n-1$ equally sized demes, smaller than the continent, called *islands*). The continent is connected with the remaining $n-1$ islands, but the islands are not connected between each other (Figure S1). Therefore, migration can only occur between the continent and the islands, but not between different islands. We order the n demes in such a way that the continent is deme number 1, whose (scaled) size is c_1. We denote c_2 the size of the other islands, and $M_1/2$ the (scaled) migration rate from the continent to each island, and $M_2/2$ the migration rate from each island to the continent. Recall that we have the following condition:

$$\forall i \in \{1, \dots n\}, \quad \sum_{j\neq i} M_{ij} c_i = \sum_{j\neq i} M_{ji} c_j. \tag{1}$$

This implies the following constraint:

$$c_1\left((n-1)\frac{M_1}{2}\right) = ((n-1)c_2)\frac{M_2}{2}$$

and thus

$$\frac{c_1}{c_2} = \frac{M_2}{M_1}. \tag{2}$$

For the case $n \geq 3$, the symmetry of the model allows us to consider, for a sample of two lineages, only five possible different configurations:

1. Both lineages are in the continent. A coalescence can occur with rate $1/c_1$, leading to configuration 5, or any of the two lineages may migrate to one of the $n-1$ islands, each with rate $M_1/2$, leading to the second configuration.

2. One lineage is in the continent and the other in an island. There can be no coalescence event, but three different migration events can occur: if the lineage in the island migrates, which arrives at rate $M_2/2$, this leads to the first configuration. The lineage in the continent can migrate at rate $M_1/2$, and it can either reach the island where the other lineage is (leading to configuration 4 below) or migrate to a different island (leading to configuration 3 below).

3. The two lineages are in different islands. No coalescence can occur and any of the two lineages can migrate to the continent, each with rate $M_2/2$, leading to configuration 2.

4. The two lineages are in the same island. Either a coalescence occurs with rate $1/c_2$, leading to configuration 5, or a migration event of one of the two lineages to the continent, each with rate $M_2/2$, leading to configuration 2.

5. The two lineages have coalesced. This is an absorbing state.

We can thus construct, for the case when $n \geq 3$, the following 5×5 transition rate matrix for a sample of size two (remembering that diagonal terms are obtained such that the sum of the the terms is zero over each row):

$$Q = \begin{pmatrix} -(1+c_1M_1(n-1))/c_1 & M_1(n-1) & 0 & 0 & 1/c_1 \\ M_2/2 & -(M_1(n-1)+M_2)/2 & (n-2)M_1/2 & M_1/2 & 0 \\ 0 & M_2 & -M_2 & 0 & 0 \\ 0 & M_2 & 0 & -M_2-1/c_2 & 1/c_2 \\ 0 & 0 & 0 & 0 & 0 \end{pmatrix}.$$

If we replace M_2 by M in equation (2) we have $M_1 = c_2M/c_1$. Then, we normalise population sizes by fixing $c_1 = 1$. Denoting $c_2/c_1 = c_2$ by c, we obtain the following transition rate matrix:

$$Q = \begin{pmatrix} -1-cM(n-1) & cM(n-1) & 0 & 0 & 1 \\ M/2 & -M(cn-c+1)/2 & (n-2)cM/2 & cM/2 & 0 \\ 0 & M & -M & 0 & 0 \\ 0 & M & 0 & -M-1/c & 1/c \\ 0 & 0 & 0 & 0 & 0 \end{pmatrix}.$$

Note that c is the ratio between the sizes of the islands and the continent, and that the diagonal entries are obtained by the constraint that the sum over each row is zero.

Figure S9 shows the IICR_s and IICR_d plots for the different sample configurations for a pair of genomes in a continent-island model with $n = 4$ (one continent and three islands). As expected from previous work on the IICR (Mazet et al., 2016; Chikhi et al., 2018), first generation hybrid individuals, whose genome is sampled in different demes, exhibit IICR plots which would be interpreted as expansions from an ancient stationary population, even though the total population size is constant. One of the most striking result is that a diploid individual sampled in one of the islands exhibits an IICR that suggests (forward in time) an ancient stationary population which first expanded before being subjected to a significant population decrease. Thus, different individuals will exhibit very different history, not because their populations were subjected to different demographic histories, but because the IICR does not represent the history of a population. It represents the coalescent history of a particular sample in a particular model.

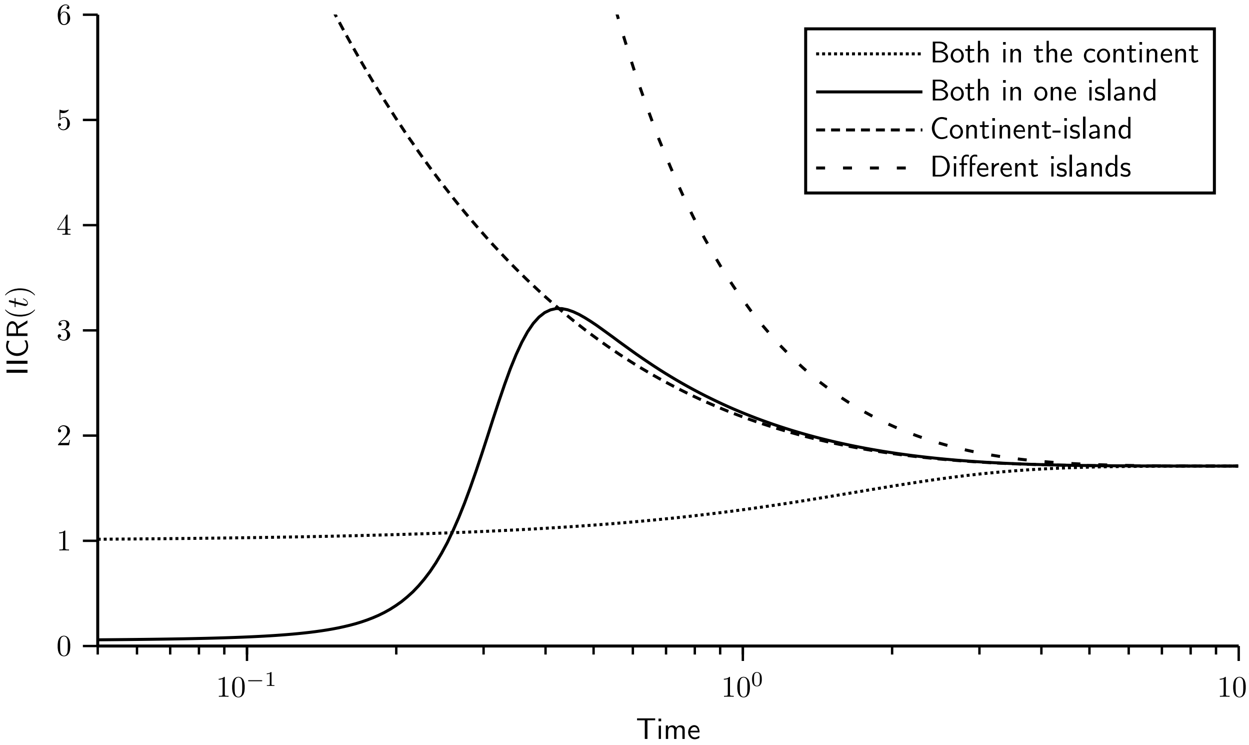

Figure S9: IICR for a continent-island model. We constructed the transition rate matrix for a model with $n = 4$, namely one continent and three same-sized islands. The sizes of the continent and of the islands were set to $c_1 = 1$ and $c_2 = 0.05$, respectively. In other words, the continent was 20 times larger than the islands. We set the migration rates to $M_1/2 = 0.05$, $M_2/2 = 1$ (note that once M_1 is set, M_2 is constrained to keep inward and outward migrant gene numbers equal, as required by equation 1). In this model there are only four types of IICR curves, two IICR_s and two IICR_d. The first two correspond to the cases where we sample the two lineages either in the continent or in one of the islands. The IICR_d curves correspond to cases where one gene comes from the continent and the other from an island or when the two genes come from two different islands.

2.2.2 Particular case: only one continent and one island

If we focus on the particular case where there is only one continent and one island (i.e. $n = 2$), then configuration 3 in the case $n \geq 3$ does not exist anymore. We thus obtain the following 4×4 transition rate matrix:

$$Q = \begin{pmatrix} -(1+c_1M_1(n-1))/c_1 & M_1(n-1) & 0 & 1/c_1 \\ M_2/2 & -(M_1+M_2)/2 & M_1/2 & 0 \\ 0 & M_2 & -M_2-1/c_2 & 1/c_2 \\ 0 & 0 & 0 & 0 \end{pmatrix}.$$

When we replace M_2 by M and c_1 by 1 as above, we get:

$$Q = \begin{pmatrix} -1-cM(n-1) & cM(n-1) & 0 & 1 \\ M/2 & -M(c+1)/2 & cM/2 & 0 \\ 0 & M & -M-1/c & 1/c \\ 0 & 0 & 0 & 0 \end{pmatrix}.$$

3 Application to real data

The mathematical tools introduced in this work allow for a very fast computation of the exact IICR of several structured scenarios. On the other hand, the IICR can be estimated from real data using the PSMC software (Li and Durbin, 2011). It is then possible to compare the PSMC curve obtained from the data (which is the IICR corresponding to the data) with the IICR that corresponds to any other structured scenario. The comparison is done by plotting together the theoretical IICR of the structured model and the IICR obtained from the data as the result of PSMC. The scripts allowing to do this comparison and some examples can be found at: https://github.com/willyrv/nssc-tools. It is then possible to do a manual exploration of the parametric space for any family of structured models under the NSSC framework and evaluate if the proposed model can explain the IICR obtained from the data. Figure S10 succinctly summarizes this process.

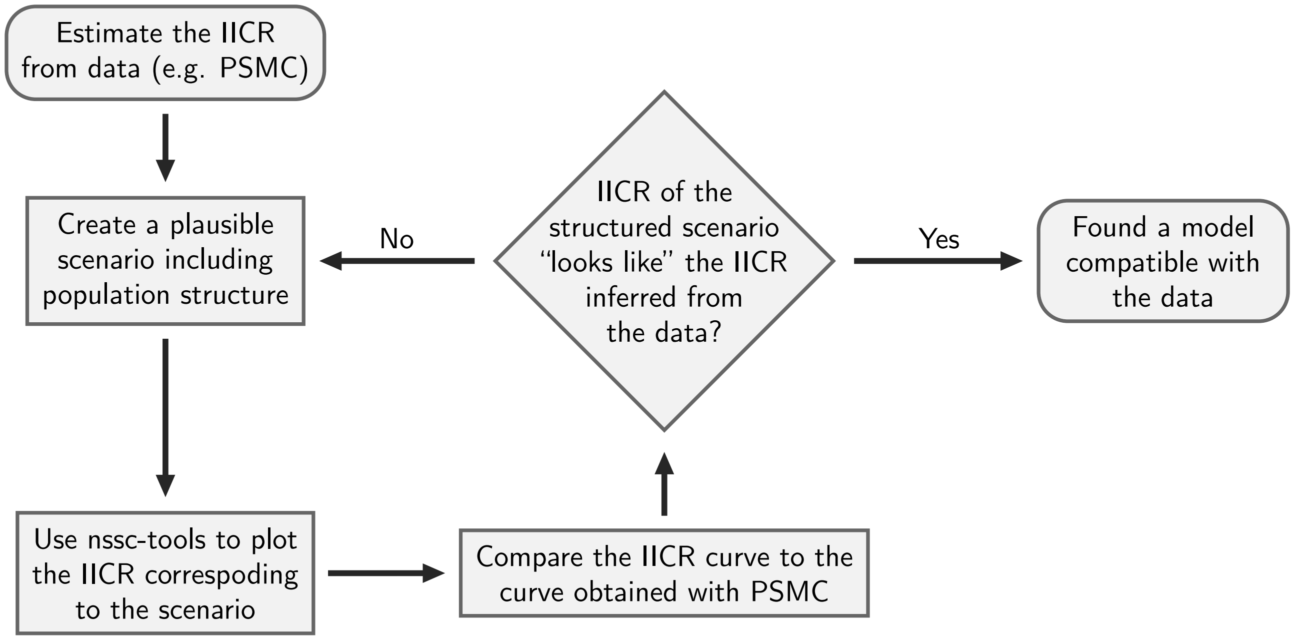

Figure S10: Schematic representation of the process of inferring structured scenarios by hand-fitting IICR curves.

References

Chikhi, L., Rodriguez, W., Grusea, S., Santos, P., Boitard, S., and Mazet, O. (2018). The IICR (inverse instantaneous coalescence rate) as a summary of genomic diversity: insights into demographic inference and model choice. *Heredity*, 120:13–24.

Kimura, M. (1953). Stepping stone model of population. *Annual Report of the National Institute of Genetics Japan*, 3:62–63.

Li, H. and Durbin, R. (2011). Inference of human population history from individual whole-genome sequences. *Nature*, 475(7357):493–496.

Malécot, G. (1948). Mathématiques de l'hérédité.

Mazet, O., Rodriguez, W., Grusea, S., Boitard, S., and Chikhi, L. (2016). On the importance of being structured: instantaneous coalescence rates and human evolution—lessons for ancestral population size inference. *Heredity*, 116(4):362–371.
